# *Staphylococcus aureus* Tissue Infection During Sepsis Is Supported by Differential Use of Bacterial or Host-Derived Lipoic Acid

**DOI:** 10.1371/journal.ppat.1005933

**Published:** 2016-10-04

**Authors:** Azul Zorzoli, James P. Grayczyk, Francis Alonzo

**Affiliations:** Department of Microbiology and Immunology, Loyola University Chicago—Stritch School of Medicine, Maywood, Illinois, United States of America; University of Tubingen, GERMANY

## Abstract

To thrive in diverse environments, bacteria must shift their metabolic output in response to nutrient bioavailability. In many bacterial species, such changes in metabolic flux depend upon lipoic acid, a cofactor required for the activity of enzyme complexes involved in glycolysis, the citric acid cycle, glycine catabolism, and branched chain fatty acid biosynthesis. The requirement of lipoic acid for metabolic enzyme activity necessitates that bacteria synthesize the cofactor and/or scavenge it from environmental sources. Although use of lipoic acid is a conserved phenomenon, the mechanisms behind its biosynthesis and salvage can differ considerably between bacterial species. Furthermore, low levels of circulating free lipoic acid in mammals underscore the importance of lipoic acid acquisition for pathogenic microbes during infection. In this study, we used a genetic approach to characterize the mechanisms of lipoic acid biosynthesis and salvage in the bacterial pathogen *Staphylococcus aureus* and evaluated the requirements for both pathways during murine sepsis. We determined that *S*. *aureus* lipoic acid biosynthesis and salvage genes exist in an arrangement that directly links redox stress response and acetate biosynthesis genes. In addition, we found that lipoic acid salvage is dictated by two ligases that facilitate growth and lipoylation in distinct environmental conditions in vitro, but that are fully compensatory for survival in vivo. Upon infection of mice, we found that *de novo* biosynthesis or salvage promotes *S*. *aureus* survival in a manner that depends upon the infectious site. In addition, when both lipoic acid biosynthesis and salvage are blocked *S*. *aureus* is rendered avirulent, implying an inability to induce lipoic acid-independent metabolic programs to promote survival. Together, our results define the major pathways of lipoic acid biosynthesis and salvage in *S*. *aureus* and support the notion that bacterial nutrient acquisition schemes are instrumental in dictating pathogen proclivity for an infectious niche.

## Author Summary

*Staphylococcus aureus* is a predominant cause of infectious diseases ranging from superficial skin and soft tissue infections to necrotizing pneumonia and sepsis. A remarkable aspect of *S*. *aureus* pathobiology lies in the ability of the microorganism to infect a wide variety of host tissues. This infectious promiscuity implies *S*. *aureus* exhibits significant adaptability when faced with disparate environments and nutritional deficiencies. In this work, we examine the mechanisms by which *S*. *aureus* acquires lipoic acid, a key cofactor involved in maintaining metabolic flux. Our studies determine that *S*. *aureus* engages in both *de novo* biosynthesis and salvage of lipoic acid in a manner that is reminiscent of pathways used by both *B*. *subtilis* and *L*. *monocytogenes* combined. Further, our work suggests that the complex mechanisms of lipoic acid acquisition dictate the range of tissues *S*. *aureus* infects and identifies a lipoic acid salvage enzyme that is dispensable for growth in vitro, but required for *S*. *aureus* pathogenesis in vivo. In sum, our results highlight the adaptability of *S*. *aureus* in the face of nutrient paucity; the importance of complex nutrient acquisition/biosynthesis pathways in promoting infection; and identify potential novel therapeutic targets that may be effective against *S*. *aureus*.

## Introduction

The survival of pathogenic microbes within host tissues depends upon the ability to adapt to the physical and nutritional restrictions imposed within that tissue. Bacteria can overcome these restrictions by stimulating or repressing metabolic gene regulatory programs; trace metal uptake and sequestration systems; metabolic cofactor biosynthesis; amino acid and sugar transport; as well as systems involved in detoxification of noxious compounds (reactive oxygen and nitrogen, organic acids, and antimicrobial peptides). The Gram-positive opportunistic pathogen *Staphylococcus aureus* causes disease in nearly all host tissues, including skin and soft tissue, bone, heart, kidney, and lungs suggesting that it uses a number of these adaptive traits to thrive in myriad nutritionally distinct environments [1, 2].

*S*. *aureus* is a leading cause of infectious disease worldwide [3–5]. The bacterium asymptomatically colonizes the anterior nares and skin of nearly 1/3 of the population and can transiently colonize many more individuals leading to a significant carrier population in communities and households [6–8]. Despite its common mode of asymptomatic colonization, upon breaching physical barriers to infection, the bacterium can disseminate widely to cause serious disease. In addition, many infectious *S*. *aureus* strains are highly resistant to antibiotics, making infections difficult to treat and increasing morbidity and mortality of disease [1, 2]. The survival of *S*. *aureus* during invasive infection is predicated on the production of major virulence factors including toxins, immunomodulatory molecules, proteases, and trace nutrient uptake systems [9–13]. Importantly, strains of *S*. *aureus* exhibit considerable genetic diversity such that infectious strains can harbor unique virulence factors and/or exhibit divergent gene regulatory schemes that preclude the development of universal therapeutic targets against all disease-causing strains [14–17]. These characteristics make the identification of universally effective antimicrobials against *S*. *aureus* a challenging pursuit.

Recently, an interest in bacterial trace nutrient acquisition has reemerged due to its important role in facilitating optimal metabolic flux during infectious disease and overcoming host nutritional immunity [18–20]. *S*. *aureus* acquires host restricted metals such as iron, manganese, and zinc to promote the activity of metabolic proteins with metal-containing enzymatic cores, thereby facilitating optimal metabolic output [21]. To overcome host-imposed nutrient restrictions, *S*. *aureus* has devised mechanisms to acquire trace metals, such as iron, during infection. Most notably, the bacterium produces siderophores and a dedicated iron scavenging and uptake system used to extract iron from its largest mammalian reservoir, heme [21–27]. In the absence of these iron-scavenging enzymes, *S*. *aureus* is severely compromised for pathogenesis [26, 28].

**Fig 1:**
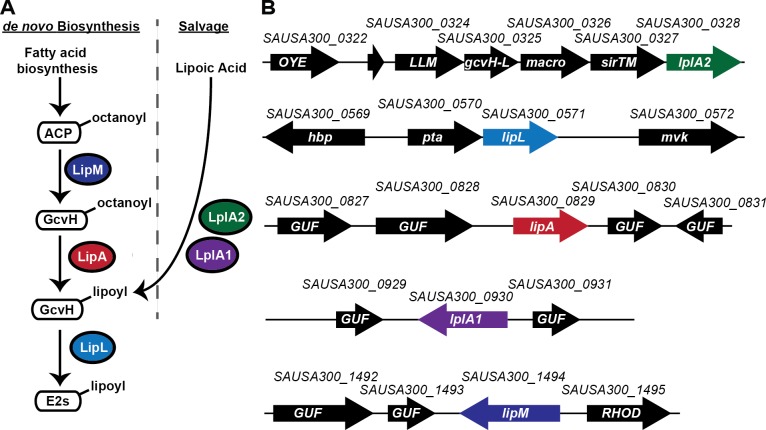
Genetic arrangement and predicted activity of putative lipoic acid biosynthesis and salvage genes in *S*. *aureus*. (A) Bacterial lipoic acid biosynthesis and salvage pathways as modeled after *B*. *subitils*. LipM–octanoyl transferase; LipA–lipoyl synthetase; LipL–lipoyl transferase; and LplA1/2 –lipoate protein ligase. (B) Gene arrangement of *S*. *aureus* putative lipoic acid biosynthesis and salvage genes. Gene designations correspond to annotations used in the genome sequence of FPR3757 USA300 (NCBI Reference Sequence: NC_007793.1). Numerical descriptors (0322–0328, 0569–0572, 0827–0831, 0929–0931, and 1492–1495) reflect the position of the indicated gene in the *S*. *aureus* genome relative to other annotated open reading frames. Colored arrows correspond to putative lipoic acid biosynthesis and salvage genes. Black arrows correspond to additional flanking genes or genes present in the operons containing *lplA2* and *lipL*. *OYE*, Old Yellow Enzyme; *LLM*, luciferase-like monooxygenase family; *gcvH-L*, glycine cleavage H-Like protein; *macro*, macrodomain protein; *sirTM*, macrodomain-linked sirtuin; *hbp*, heme binding protein; *pta*, phosphotransacetylase; *mvk*, mevalonate kinase; *GUF*, gene of unknown function; and *RHOD*, rhodanase domain-containing protein. One additional small putative open reading frame exists directly upstream of *LLM* and is designated with a short black arrow.

Along with trace metals, *S*. *aureus* and other infectious microbes require additional cofactors to maintain metabolic flux in disparate environments. One such cofactor is lipoic acid. Lipoic acid is an enzyme complex cofactor intimately linked to intermediary metabolism [29]. It is found in all kingdoms of life including bacteria, yeast, and higher order eukaryotes, though the mechanisms involved in its biosynthesis and salvage show considerable diversity [29, 30]. It is a sulfur-containing molecule that is covalently linked to proteins in large multi-subunit enzymatic complexes and is involved in redox coupling during oxidative and one carbon metabolism [31]. The most well-known lipoic acid-containing enzymes include the pyruvate dehydrogenase (PDH), 2-oxoglutarate dehydrogenase (OGDH), branched-chain 2-oxoacid dehydrogenase (BCODH) complexes, and the glycine cleavage system (Gcs) [29, 30]. Lipoyl moieties are found covalently linked to a conserved lysine within the “E2” or “H” subunit of these complexes. Bacteria acquire lipoic acid by one of two mechanisms: *de novo* biosynthesis or salvage from the environment [29]. The mechanisms by which synthesis and salvage occur are not conserved in all bacteria, nor is lipoic acid necessarily required for cellular viability (e.g., *Helicobacter pylori*–due to its use of alternative non-lipoylated metabolic enzymes) [30, 32–35]. Central to all *de novo* biosynthesis pathways is the lipoic acid synthetase, LipA [29, 36]. LipA is responsible for the insertion of two sulfur atoms into the precursor molecule octanoic acid to generate lipoic acid (Fig 1A) [37]. The synthetase is broadly conserved in both pathogenic and non-pathogenic *Firmicutes* and serves as a primary indicator of a functional *de novo* lipoic acid biosynthesis pathway [30]. Lipoic acid synthetase activity is preceded by an octanoyl transferase that uses amidotransferase functions to shuttle octanoic acid, derived from fatty acid biosynthesis, directly to a conserved lysine of the protein to be lipoylated (Fig 1A) [38, 39]. A comparison to known enzymes in *Bacillus subtilis* indicates that *S*. *aureus* contains one gene that encodes a predicted octanoyl transferase (*SAUSA300_1494—lipM*) and another that encodes a putative lipoyl transferase (*SAUSA300_0571—lipL*) (Table 1 and Fig 1A) [39–41]. *S*. *aureus* also contains two genes encoding predicted ligases with presumptive roles in lipoic acid salvage (*SAUSA300_0930 –lplA1* and *SAUSA300_0328 –lplA2*) (Fig 1A) [30, 41, 42]. Recent biochemical studies suggest that the two ligases may have preferred targets for lipoylation, however their precise roles in lipoic acid salvage and their use of alternative substrates have not been established [42]. Aside from *S*. *aureus* and *B*. *anthracis*, no other pathogenic *Firmicutes* appear to encode the diversity of enzymes involved in both *de novo* lipoic acid biosynthesis and salvage [30]. For example, while nearly all staphylococcal species, including the pathogenic *Staphylococcus epidermidis*, *Staphylococcus haemolyticus*, and *Staphylococcus lugdunensis*, contain genes encoding enzymes necessary for *de novo* biosynthesis, only *S*. *aureus* harbors two ligases in addition to its *de novo* biosynthesis genes. This implies that *S*. *aureus* and *B*. *anthracis* use more complex lipoic acid acquisition schemes compared to that of other pathogenic *Firmicutes*.

**Table 1:** Putative lipoic acid biosynthesis and salvage enzymes in *S*. *aureus*.

*S*. *aureus* Annotation	Gene Designation	Protein Name	Predicted function/ % amino acid identity compared to *B*. *subtilis*	Predicted function/ % amino acid identity compared to *L*. *monocytogenes*
**Synthetase**	*SAUSA300_0829*	LipA	Lipoate synthetase–LipA (79%)	NA
**Ligase**	*SAUSA300_0571*	LipL	Lipoyl transferase–LipL (54%)	NA
**Ligase**	*SAUSA300_1494*	LipM	Octanoyl transferase–LipM (62%)	NA
**Ligase**	*SAUSA300_0930*	LplA1	Lipoate ligase–LplJ (57%)	Lipoate ligase—LplA1 (53%) LplA2 (47%)
**Ligase**	*SAUSA300_0328*	LplA2	Lipoate ligase–LplJ (39%)	Lipoate ligase—LplA1 (41%) LplA2 (37%)

NA–Not present in *L*. *monocytogenes* (LipM and LipA) or not assessed (LipL)

Lipoic acid salvage has thus far been linked to the virulence of a small number of pathogens [30, 43–47]. A role for lipoic acid acquisition in promoting the ability to adapt to nutrient restriction is most well studied in *Listeria monocytogenes*. *L*. *monocytogenes* is a lipoic acid auxotroph that produces two lipoic acid salvage enzymes, LplA1 and LplA2, but no *de novo* biosynthesis enzymes [30, 45, 48]. LplA1 is dispensable for growth in broth culture, but is essential for *L*. *monocytogenes* replication in the host cytosol [48]. LplA1 is thought to facilitate scavenging of lipoic acid from host lipoylated peptides, thereby allowing the bacterium to acquire the cofactor in vivo, where free lipoic acid is limiting [48, 49]. In contrast, LplA2 is dispensable for lipoic acid salvage in vivo [48]. Thus, lipoic acid acquisition in *L*. *monocytogenes* is contextual and relies on dedicated ligases for salvage during infection. *Chlamydia trachomatis* is also believed to use salvage mechanisms for survival in vivo, although it is not known whether *de novo* biosynthesis also plays a role [50]. In other non-bacterial pathogens, such as *Plasmodium* (spp.), the LplA2 orthologue is dispensable in vivo, but the ligase, LplA1, is critical for survival of the parasite during expansion within red blood cells [46].

In summary, it is becoming evident that the mechanisms of lipoic acid salvage and biosynthesis may play critical roles in the ability of bacteria to adapt to lipoic acid restriction in vivo. *S*. *aureus* is unique in that it can colonize nearly all host tissues, most of which are likely to have varied, albeit universally low, levels of bioavailable free lipoic acid [51]. In this work, we show that *S*. *aureus* uses its complex lipoic acid biosynthesis and salvage pathways to promote optimal metabolic efficiency, and thus viability, in vitro and during infection. We first decipher the mechanisms of lipoic acid biosynthesis and salvage and use mutants in either pathway to interrogate their functional roles during invasive infection. To our surprise, we find condition-specific dependency on individual salvage enzymes for survival and lipoylation in vitro and define requirements for both *de novo* biosynthesis and salvage in vivo that depends on the site of infection. Further, the genetic arrangement of the lipoic acid biosynthesis and salvage genes in *S*. *aureus* highlights novel associations with fermentative metabolism and responses to reactive oxygen species that have not been appreciated for other Gram-positive pathogens. Together, our results allude to a novel mechanism by which lipoic acid biosynthesis and salvage activities are coordinated in *S*. *aureus* to facilitate unrestricted tissue infection capabilities.

## Results

### *S*. *aureus* contains five genes that encode putative lipoic acid biosynthesis and salvage enzymes

Based upon amino acid sequence identity comparisons to *B*. *subtilis* and *L*. *monocytogenes*, we identified five *S*. *aureus* open reading frames within the sequenced genome of USA300 isolate FPR3757 that encode proteins with similarities to both lipoic acid biosynthesis and salvage enzymes (Table 1 and S1 Fig). One gene (*SAUSA300_0829*—*lipA*) encodes a putative lipoic acid synthetase, LipA, with 79% amino acid sequence identity to *B*. *subtilis* LipA. The remaining genes (*SAUSA300_0328*, *SAUSA300_0571*, *SAUSA300_0930*, and *SAUSA300_1494*) were all previously annotated as members of the lipoate protein ligase family, PFAM03099, a highly divergent family of proteins that can exhibit activities associated with both *de novo* biosynthesis or lipoic acid salvage [39–41]. Amino acid sequence alignments of SAUSA300_0571 showed 54% identity to the *B*. *subtilis* lipoyl transferase, LipL, while SAUSA300_1494 had 62% identity to LipM, the octanoyl transferase required for *de novo* biosynthesis of lipoic acid in *B*. *subtilis* [39, 40]. In contrast, SAUSA300_0930 and SAUSA300_0328 are 57% and 39% identical respectively to the sole lipoic acid ligase of *B*. *subtilis*, LplJ, suggesting a potential role for these two proteins in *S*. *aureus* lipoic acid salvage [41]. To further investigate this possibility, we also compared amino acid sequence identity of SAUSA300_0930 and SAUSA300_0328 to *L*. *monocytogenes* lipoic acid salvage enzymes LplA1 and LplA2 [48]. We found that SAUSA300_0930 has 53% and 47% identity to LplA1 and LplA2 respectively, while SAUSA300_0328 has 41% and 37% identity. Furthermore, recent studies have determined both SAUSA300_0930 and SAUSA300_0328 function as lipoic acid ligases that catalyze the addition of free lipoic acid to two glycine cleavage H (GcvH) proteins (GcvH and GcvH-L) in *S*. *aureus* [42]. Based upon these amino acid sequence identity comparisons to lipoic acid biosynthesis and salvage enzymes in *B*. *subtilis* and *L*. *monocytogenes*, as well as naming conventions implemented by Rack *et al*, we designate the *S*. *aureus* lipoic acid biosynthesis and salvage proteins as follows: SAUSA300_0829 –LipA, SAUSA300_0571 –LipL, SAUSA300_1494 –LipM, SAUSA300_0930 –LplA1, and SAUSA300_0328 –LplA2 (Table 1 and Fig 1B) [42].

### Lipoic acid biosynthesis and salvage genes are widely spread throughout the *S*. *aureus* genome

To ascertain the genetic arrangement of the lipoic acid biosynthesis and salvage genes in *S*. *aureus* we evaluated both the location and gene content flanking each biosynthesis and salvage open reading frame. *lipA*, *lplA1*, and *lipM* consist of single open reading frames and are separated from each other by at least 100 genes (Fig 1B). The genes are dispersed throughout the *S*. *aureus* chromosome and thus do not appear to be linked in any capacity. In contrast, *lplA2* and *lipL* are each associated with a putative operon. *lplA2* is in an operon with genes encoding a macrodomain linked sirtuin (SirTM) and a GcvH-like protein (GcvH-L) [42]. The product of *lplA2* was previously shown to lipoylate GcvH-L, a modification that permits ADP ribosylation of GcvH-L by SirTM [42]. Lipoylation followed by ADP-ribosylation of GcvH-L is hypothesized to promote *S*. *aureus* resistance to redox stress, although this activity has not yet been confirmed. *lipL* exists in a putative operon with the *pta* gene of *S*. *aureus* (Fig 1B). *pta* encodes the phosphotransacetylase enzyme component of the phosphotransacetylase-acetate kinase (Pta-AckA) pathway, involved in the generation of ATP via acetate biosynthesis during growth in the presence of glucose and oxygen [52–55]. To our knowledge this is the first identified genetic association of a lipoic acid biosynthesis gene with a gene directly involved in acetate biosynthesis. In all cases, the lipoic acid biosynthesis and salvage genes of *S*. *aureus* show a high degree of conservation among *S*. *aureus* lineages and do not appear to be encoded within mobile elements. Together, these observations indicate *S*. *aureus* lipoic acid biosynthesis and salvage genes are conserved and broadly distributed throughout the *S*. *aureus* genome. In addition, *lipL* and *lplA2* exhibit novel associations with metabolic and stress response genes respectively.

### LipA, LipM and LipL are *de novo* biosynthesis enzymes involved in lipoic acid synthesis, octanoyl transfer, and lipoyl transfer

**Fig 2:**
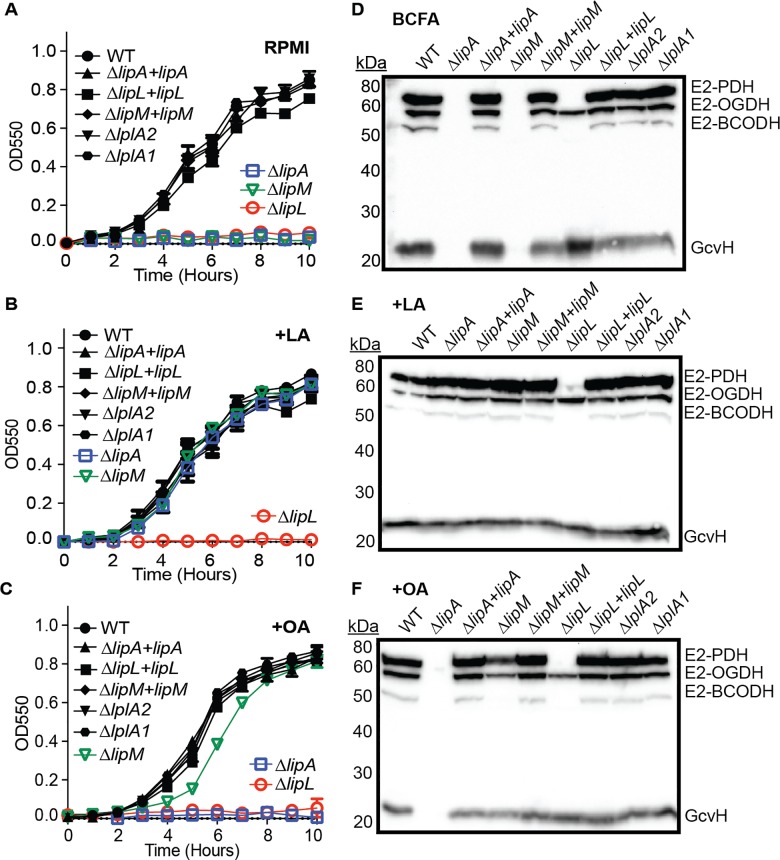
Deletion of *lipA*, *lipM*, or *lipL* renders *S*. *aureus* auxotrophic for lipoic acid. (A-C) Growth curves of the indicated strains in (A) RPMI, (B) RPMI + 25 nM lipoic acid (LA) or (C) RPMI + 250 μM octanoic acid (OA). (D-F) Whole cell lysates of *S*. *aureus* collected after 9 hours of growth in (D) RPMI + BCFA (2-methyl butyric acid, isovaleric acid, isobutyric acid, and sodium acetate), (E) RPMI + BCFA + 5 μM lipoic acid (LA), or (F) RPMI + BCFA + 175 μM octanoic acid (OA) followed by immunoblotting for lipoic acid-containing proteins. In all growth curves, the mean +/- standard deviation of triplicate measures is shown. In any case where an error bar is not visible, the standard deviation was smaller than the size of the symbol used at that data point.

To ascertain the functions of the enzymes involved in lipoic acid biosynthesis and salvage in *S*. *aureus*, we generated a series of in-frame deletion mutants in each of the 5 putative lipoic acid biosynthesis genes (Δ*lipA*, Δ*lipM*, Δ*lipL*, Δ*lplA1*, and Δ*lplA2*) and evaluated whether or not gene deletion rendered *S*. *aureus* auxotrophic for lipoic acid (LA) or octanoic acid (OA), the precursor of lipoic acid. We found that Δ*lipA*, Δ*lipM*, and Δ*lipL* mutants are unable to replicate in defined medium (RPMI) lacking exogenous lipoic acid or octanoic acid (Fig 2A). Single copy complementation of each mutant with *lipA*, *lipM*, and *pta-lipL* (designated *lipL* in all figures) under control of the constitutive *P*_*HELP*_ promoter restored normal growth kinetics to all strains (Fig 2A). Notably, complementation of Δ*lipL* required introduction of the entire *pta-lipL* operon into the Δ*lipL* strain. Attempts at complementation with *lipL* or *pta* alone were unsuccessful (S2 Fig), an outcome we suspect is due to co-translational coupling of *pta* and *lipL* (see Discussion). In contrast to RPMI alone, Δ*lipA* and Δ*lipM* grew normally after supplementation with lipoic acid, while the growth of Δ*lipM* was restored with octanoic acid supplementation (Fig 2B and 2C). In these studies, higher concentrations of octanoic acid (175–250 μM) were required to stimulate growth and detectable lipoylation compared to lipoic acid (0.025 μM– 5 μM). This is similar to prior studies in *E*. *coli*, where supplementation of octanoic acid at 50 μM was required to stimulate growth and lipoylation of *E*. *coli lipB* mutants compared to 0.025 μM lipoic acid [59, 60]. A Δ*lipL* mutant was unable to grow in all conditions, regardless of lipoic acid or octanoic acid supplementation (Fig 2A–2C). Δ*lplA1* and Δ*lplA2* mutants had growth patterns that were indistinguishable from WT in all conditions, suggesting these genes are not involved in *de novo* biosynthesis of lipoic acid. Collectively, these data indicate Δ*lipA*, Δ*lipM*, and Δ*lipL* mutants are auxotrophic for lipoic acid; Δ*lipM* is auxotrophic for octanoic acid; and Δ*lplA1* and Δ*lplA2* are not required for *de novo* biosynthesis of lipoic acid.

To evaluate the lipoylation capabilities of these mutants, we grew all strains in a modified bypass medium previously used to support the growth of *B*. *subtilis* lipoic acid auxotrophs [41, 61]. RPMI medium was supplemented with the branched chain carboxylic acids (BCFA), isovalerate, isobutyrate, and 2-methyl butyrate along with sodium acetate in order to bypass the essential requirements of PDH and BCODH products for growth in broth culture (S3 Fig). Strains were subcultured into bypass medium (BCFA) or BCFA supplemented with lipoic acid (LA) and octanoic acid (OA) and the fate of either substrate was determined by performing anti-lipoic acid immunoblots on whole cell lysates obtained from early stationary phase cultures (~9 hours) (Fig 2D–2F and S4 Fig). In *S*. *aureus*, five metabolic protein complex components contain conserved lipoyl domains (NCBI: cd06849) with target lysine residues for lipoylation [30]. These proteins include the E2 subunits of the PDH, OGDH, and BCODH complexes; the H protein of the glycine cleavage system; and the GcvH-like protein GcvH-L. Despite encoding 5 putative lipoyl domain-containing proteins, an anti-lipoic acid immunoblot of *S*. *aureus* whole cell lysates identified four lipoylated molecular weight species that we suspect corresponded to the three E2 and one H (GcvH) components (S5 Fig). To determine the identity of the lipoylated species observed in immunoblots, we first generated deletion mutants of the genes encoding E2-PDH (*SAUSA300_0995)*, E2-OGDH (*SAUSA300_1305)*, and GcvH (*SAUSA300_0791)* in *S*. *aureus* strain LAC. We identified E2-PDH and E2-OGDH as the two largest molecular weight species and GcvH as the smallest (S5 Fig). Although numerous attempts were made to generate an *e2-BCODH* (*SAUSA300_1464)* mutant, we were unsuccessful despite carrying out all mutagenesis steps in BCFA-containing medium. Although unclear at this time, we suspect an *e2-BCODH* mutant is compromised for growth to a point that prohibits its isolation using standard mutagenesis techniques (see Materials and Methods). Nonetheless, we can infer that E2-BCODH corresponds to the third largest molecular weight species since all other bands were successfully identified and molecular weight predictions coincide with the E2 subunit of the BCODH complex. The GcvH-like protein, GcvH-L, is presumed to be upregulated under redox stress conditions, therefore we suspect this may be the reason it is undetectable under the growth conditions used in this study [62, 63].

Consistent with our assessment of growth in RPMI without supplements, immunoblots of whole cell lysates of BCFA-grown Δ*lipA* and Δ*lipM* mutants lack all lipoylated proteins (Fig 2D). In contrast, although unable to replicate in RPMI, when grown in BCFA medium a Δ*lipL* mutant is able to lipoylate both GcvH and E2-OGDH, but not E2-PDH and E2-BCODH (Fig 2D). Upon supplementation with exogenous lipoic acid Δ*lipA* and Δ*lipM* exhibited complete restoration of lipoylation, while the Δ*lipL* mutant remained incapable of lipoylating E2-PDH and E2-BCODH (Fig 2E). When octanoic acid was supplemented, Δ*lipM* displayed partial restoration of lipoylation, albeit at reduced efficiency (Fig 2F). Loading 1.5 times more sample volume allowed full visualization of all four E2 and H proteins with the Δ*lipM* mutant grown in BCFA+OA (S6 Fig). In all cases, Δ*lplA1* and Δ*lplA2* mutants had lipoylation patterns that were indistinguishable from WT (Fig 2D–2F). Together these results suggest LipA, LipM, and LipL are each critical for the *de novo* biosynthesis of lipoic acid. LipA behaves as a lipoic acid synthetase; LipM as a likely octanoyl transferase that acts upstream of LipA; and LipL as a likely amidotransferase (lipoyl transferase) capable of facilitating lipoylation of E2-PDH, and E2-BCODH. In contrast, LplA1 and LplA2 do not appear to be involved in *de novo* biosynthesis of lipoic acid and presumably have roles in lipoic acid/octanoic acid salvage.

### *S*. *aureus* uses *de novo* biosynthesis of lipoic acid for infection of the heart, but not the kidney, during bloodstream infection

**Fig 3:**
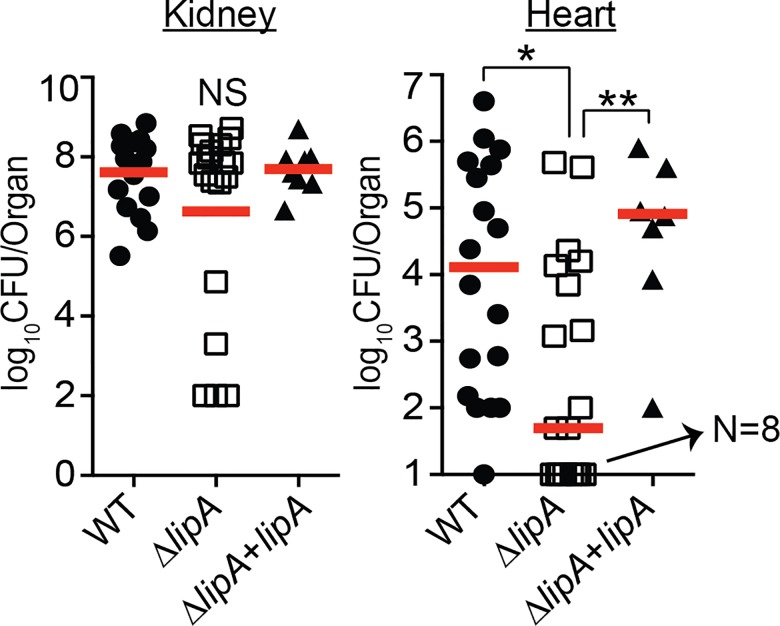
*de novo* synthesis of lipoic acid is critical for infection of the heart, but not the kidney of mice after bloodstream infection. Bacterial burden in kidneys and hearts of mice 96 hours after infection via the bloodstream with 1.0 x 10^7^ CFU WT, Δ*lipA*, and Δ*lipA+lipA*. log_10_CFU per organ is displayed for each infected mouse along with the median as a measure of central tendency. Animal numbers displayed are as follows: WT, *N* = 18; Δ*lipA*, *N* = 19; Δ*lipA+lipA*, *N* = 8. For animals infected with WT, 1 mouse had undetectable CFU recovered from the heart, whereas *N* = 8 mice had undetectable CFU recovered from the hearts after infection with Δ*lipA*. Statistics were calculated using a nonparametric 1-way ANOVA with Kruskal-Wallis multiple comparisons post-test to evaluate statistical significance between groups. In the heart, statistically significant differences were observed for WT compared to Δ*lipA*, *, *P*<0.05; and Δ*lipA* compared to Δ*lipA+lipA*, *P*<0.01. No statistically significant differences in CFU were observed in the kidney.

Thus far, we have determined that LipA behaves as a lipoic acid synthetase in *S*. *aureus* that is required for *de novo* biosynthesis of lipoic acid. As such, a Δ*lipA* mutant relies entirely on salvage of exogenous lipoic acid or its precursors in order to replicate in vitro. We reasoned that if *S*. *aureus* were to be introduced into similar lipoic acid-limiting environments in vivo (nearly all lipoic acid is protein bound, while free lipoic acid has a very short half-life) [64], then survival of the bacterium during infection would necessarily require LipA. To test whether or not this was the case, we used a murine *S*. *aureus* bloodstream infection model to assess the requirement for lipoic acid biosynthesis during infection of host tissues. We infected 4–6 week old female ND4 Swiss Webster mice with 1x10^7^ CFU of WT, Δ*lipA*, or Δ*lipA*+*lipA* via the retro-orbital venous plexus to induce sepsis. In this model, both the kidney and heart are infected by *S*. *aureus* at 96 hours post-infection [28]. After 96-hours, we removed kidneys and hearts, homogenized the tissues, and quantified the number of bacteria present at each infectious site (Fig 3). Remarkably, we found that the bacterial burden in kidneys was similar for all strains tested (Fig 3). In contrast, bacterial burden in the heart was reduced by greater than two logs for the Δ*lipA* mutant (Fig 3). Together, these findings suggest that *de novo* biosynthesis of lipoic acid is critical for infection of certain tissue sites (heart) but not others (kidney), and implies that mechanisms of lipoic acid salvage may compensate for a lack of *de novo* lipoic acid biosynthesis, but only in tissues where the cofactor is more readily accessible (kidney).

### LplA1 is the primary lipoic acid salvage enzyme used during in vitro growth

**Fig 4:**
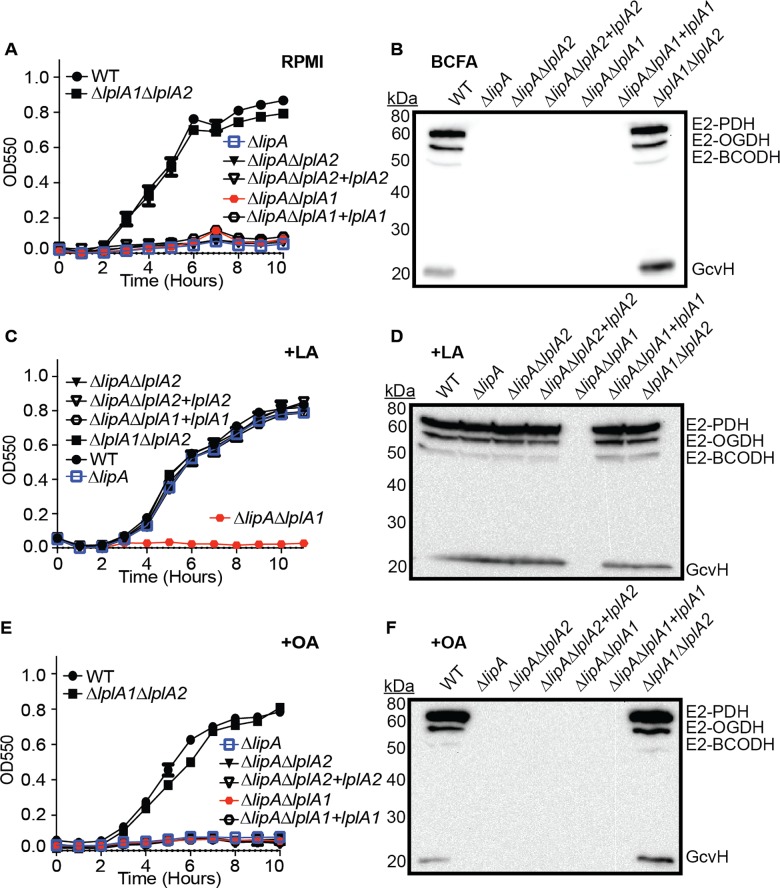
The LplA1 ligase is necessary for salvage of lipoic acid during in vitro growth. (A, C, and E) Growth curves of the indicated strains in (A) RPMI, (C) RPMI + 25 nM lipoic acid (LA) or (E) RPMI + 250 μM octanoic acid (OA). (B, D, and F) Whole cell lysates of *S*. *aureus* collected after 9 hours of growth in (B) RPMI + BCFA (2-methyl butyric acid, isovaleric acid, isobutyric acid, and sodium acetate), (D) RPMI + BCFA + 5 μM lipoic acid (LA), or (F) RPMI + BCFA + 175 μM octanoic acid (OA) followed by immunoblotting for lipoic acid-containing proteins. In all growth curves, the mean +/- standard deviation of triplicate measures is shown. In any case where an error bar is not visible, the standard deviation was smaller than the size of the symbol used at that data point.

Thus far, our data and the data of Rack *et al* suggest that LplA1 and LplA2 are *S*. *aureus* lipoic acid ligases [42]. To directly test whether LplA1 and LplA2 behave as ligases, we generated a set of *lplA1* and *lplA2* deletion mutants in a Δ*lipA* mutant background in order to block *de novo* biosynthesis and facilitate assessment of lipoic acid salvage activities. The following panel of mutant and complementation strains were generated: Δ*lipA*Δ*lplA1*, Δ*lipA*Δ*lplA2*, Δ*lplA1*Δ*lplA2*, Δ*lipA*Δ*lplA1*+*lplA1*, and Δ*lipA*Δ*lplA2*+*lplA2*. We assessed growth characteristics in RPMI +/- lipoic acid and octanoic acid and performed anti-lipoic acid immunoblots on whole cell lysates as described above (Fig 4 and S4 Fig). Consistent with our previous results that identified LipA as a lipoic acid synthetase, all strains carrying a Δ*lipA* mutation were unable to replicate in RPMI and unable to lipoylate proteins when grown in BCFA bypass medium (Fig 4A and 4B). Identical results were seen for octanoic acid supplementation (Fig 4E and 4F). When lipoic acid was supplemented into the medium, all strains were able to grow except for the Δ*lipA*Δ*lplA1* double mutant (Fig 4C). In addition, this strain was unable to use exogenous lipoic acid present in the culture medium to lipoylate E2 and H proteins (Fig 4D). In contrast, the Δ*lipA*Δ*lplA2* double mutant grew normally when lipoic acid was present in the culture medium (Fig 4C). These data suggest that LplA1 is required for lipoic acid salvage in vitro, while a role for LplA2 in lipoic acid salvage was not uncovered in these conditions.

### Alterations to the in vitro growth environment permit lipoic acid ligation by LplA2

**Fig 5:**
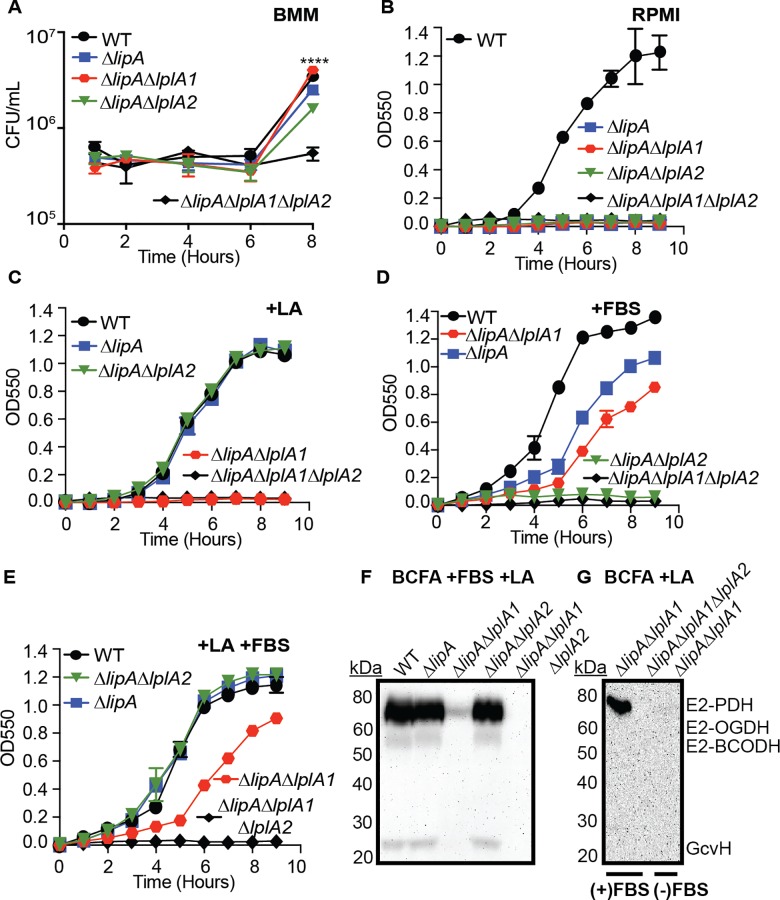
Lipoic acid salvage enzymes differentially stimulate bacterial growth and lipoylation in a growth condition-dependent manner. (A) Survival and outgrowth of *S*. *aureus* after infection of murine bone marrow derived macrophages at a multiplicity of infection of one. (B-E) Growth curves of the indicated strains in (B) RPMI, (C) RPMI + 25 nM lipoic acid (LA), (D) RPMI + 20% FBS, or (E) RPMI + 25 nM lipoic acid + 20% FBS. (F-G) Whole cell lysates of *S*. *aureus* collected after 9 hours of growth in (F) RPMI + BCFA (2-methyl butyric acid, isovaleric acid, isobutyric acid, and sodium acetate) + 20% FBS + 5 μM lipoic acid (LA), or (G) RPMI + BCFA + 5 μM LA (samples from lanes 1 and 2 were derived from strains grown in medium supplemented with additional 20% FBS, while samples in lane 3 were derived from strains grown in BCFA+LA only) followed by immunoblotting for lipoic acid-containing proteins. In all growth curves, the mean +/- standard deviation of triplicate measures is shown. In any case where an error bar is not visible, the standard deviation was smaller than the size of the symbol used at that data point. Statistical significance in (A) was determined for all strains compared to Δ*lipA*Δ*lplA1*Δ*lplA2* using Two-way ANOVA with Tukey’s post-test. ****, *P*<0.0001.

Thus far our findings suggest that LplA1, but not LplA2 is necessary for lipoic acid acquisition in broth culture. In the pathogenic bacterium *L*. *monocytogenes*, which harbors two lipoic acid ligases, LplA1 is required for the use of host derived lipoyl peptides and promotes survival in the host cell cytosol, while LplA2 permits use of free lipoic acid and growth in broth culture [48]. In *S*. *aureus*, recombinant LplA1 and LplA2 are capable of using free lipoic acid for lipoylation of recombinant GcvH and GcvH-L in vitro [42]. In addition, the operon-encoded LplA2 is induced by environmental stress conditions that resemble the host environment [62, 63]. Given this information we reasoned that LplA2 in *S*. *aureus* might exhibit dependency on growth conditions or nutrient availabilities imposed by host cells. Thus, we evaluated the ability of *S*. *aureus* Δ*lipA*Δ*lplA1* and Δ*lipA*Δ*lplA2* double mutants to survive and escape from within murine macrophages. We infected primary bone marrow derived macrophages at a multiplicity of infection of one with WT, Δ*lipA*, Δ*lipA*Δ*lplA1*, Δ*lipA*Δ*lplA2*, and Δ*lipA*Δ*lplA1*Δ*lplA2 S*. *aureus* strains for 30 minutes followed by gentamicin treatment to kill extracellular bacteria and monitored bacterial survival and escape/outgrowth over time. For the first six hours post-infection, all strains remained cytosolic with limited replication within the macrophage (Fig 5A). At 8 hours post-infection macrophages began to lyse leading to rapid replication of WT, Δ*lipA*, Δ*lipA*Δ*lplA1*, and Δ*lipA*Δ*lplA2* strains, but not the Δ*lipA*Δ*lplA1*Δ*lplA2* triple mutant, in the culture medium. These data suggest that all *S*. *aureus* strains are able to survive equally well within primary murine macrophages. Furthermore, upon bone marrow macrophage lysis, either LplA1 or LplA2 is sufficient to stimulate bacterial outgrowth in a manner that is dependent on lipoic acid salvage.

We reasoned the rescued growth of a Δ*lipA*Δ*lplA1* mutant in the presence of bone marrow derived macrophages was either due to the acquisition of alternative lipoyl substrates by LplA2 that were released by the macrophage, or the result of *lplA2* induction within the culture medium leading to subsequent incorporation of free lipoic acid. The base medium used to culture bone marrow macrophages (DMEM) does not contain free lipoic acid, however the medium is supplemented with 20% fetal bovine serum (FBS) to support cell viability. Therefore we tested whether FBS was sufficient to induce growth of the Δ*lipA*Δ*lplA1* double mutant in vitro. We performed growth curves using standard lipoic acid-deficient growth medium (RPMI), RPMI supplemented with lipoic acid, RPMI supplemented with 20% FBS, or RPMI + lipoic acid + 20% FBS and monitored growth of WT, Δ*lipA*, Δ*lipA*Δ*lplA1*, Δ*lipA*Δ*lplA2*, and Δ*lipA*Δ*lplA1*Δ*lplA2 S*. *aureus* (Fig 5B–5E). Consistent with our earlier assessment, only WT *S*. *aureus* grew in RPMI medium without lipoic acid (Fig 5B). When supplemented with lipoic acid, Δ*lipA*, and Δ*lipA*Δ*lplA2* grew identically to the WT strain, while Δ*lipA*Δ*lplA1*, and Δ*lipA*Δ*lplA1*Δ*lplA2* did not (Fig 5C). In contrast, upon supplementation with 20% FBS Δ*lipA* and Δ*lipA*Δ*lplA1* were partially restored for growth, while Δ*lipA*Δ*lplA2* was not (Fig 5D). Additional supplementation of RPMI + 20% FBS with exogenous lipoic acid fully restored the growth of Δ*lipA* and Δ*lipA*Δ*lplA2*, whereas Δ*lipA*Δ*lplA1* remained partially restored (Fig 5E). Additionally, evaluation of lipoylation by immunoblot using BCFA bypass medium supplemented with 20% FBS and lipoic acid demonstrated normal lipoylation for WT, Δ*lipA*, and Δ*lipA*Δ*lplA2* strains and partial lipoylation of E2 PDH by the Δ*lipA*Δ*lplA1* double mutant (Fig 5F). To determine if additional E2 or H subunits were lipoylated at low levels in the Δ*lipA*Δ*lplA1* mutant we performed a second immunoblot with 2.5X more sample volume and found that in the presence of FBS only lipoylation of E2-PDH was detectable (Fig 5G). Furthermore, the lipoylation of E2-PDH depended entirely on growth in the presence of serum as growth in BCFA without FBS showed no lipoylation (Fig 5G). Lipoyl E2-PDH was not a contaminant derived from serum supplementation as a Δ*lipA*Δ*lplA1*Δ*lplA2* triple mutant, grown in the presence of serum, showed no lipoyl proteins by immunoblot (Fig 5F and 5G). Notably, growth of all strains in serum altered lipoylation profiles to the extent that only E2-PDH, E2-OGDH, and GcvH were detectable, but not E2-BCODH (Fig 5F and 5G). Further, the relative abundance of E2-PDH was substantially higher than that of E2-OGDH and GcvH (Fig 5F and 5G). All together these data demonstrate that: (1) LplA2 is sufficient to support limited lipoic acid utilization and bacterial growth in vitro upon supplementation of FBS; (2) specific conditions can be identified that promote LplA2 function; (3) LplA1 is not sufficient for growth in serum and only promotes growth in the presence of free lipoic acid suggesting the lipoyl substrate in serum is not free lipoic acid, or that *lplA1* gene expression/activity is induced upon addition of excess lipoic acid; and (4) under the described in vitro conditions the lipoylation capacity of LplA2 thus far appears restricted to E2-PDH.

### LplA1 and LplA2 contribute to lipoic acid salvage during infection and promote optimal infection efficiency at sites where *de novo* biosynthesis is dispensable

**Fig 6:**
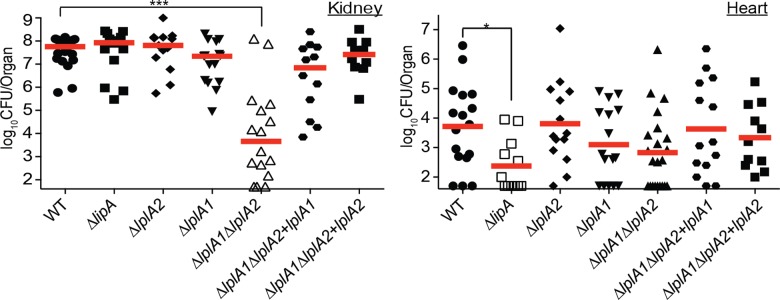
Lipoic acid salvage mechanisms are critical for infection of the kidney and require either LplA1 or LplA2. Bacterial burden in kidneys and hearts of mice 96 hours after infection via the bloodstream with 1.0 x 10^7^ CFU WT, Δ*lipA*, Δ*lplA1*, Δ*lplA2*, Δ*lplA1*Δ*lplA2*, Δ*lplA1*Δ*lplA2+lplA1*, and Δ*lplA1*Δ*lplA2*+*lplA2*. log_10_CFU per organ is displayed for each infected mouse along with the median as a measure of central tendency. Animal numbers are as follows: WT, *N* = 20; Δ*lipA*, *N* = 15; Δ*lplA1*, *N* = 15; Δ*lplA2*, *N* = 14, Δ*lplA1*Δ*lplA2*, *N* = 16; Δ*lplA1*Δ*lplA2+lplA1*, *N* = 12; Δ*lplA1*Δ*lplA2+lplA2*, *N* = 12. Statistics were calculated using a nonparametric 1-way ANOVA with Kruskal-Wallis multiple comparisons post-test to evaluate statistical significance between groups. In the kidney, statistically significant differences were observed when comparing Δ*lplA1*Δ*lplA2* to WT, Δ*lipA*, and Δ*lplA2* ****, *P*<0.0001; and Δ*lplA1*Δ*lplA2* to Δ*lplA1* and Δ*lplA1*Δ*lplA2+lplA2* *, *P*<0.05. In the heart, statistically significant differences in CFU were observed for only WT compared to Δ*lipA* *, *P*<0.05.

Our in vitro assessment of lipoic acid ligase function suggests that LplA1 is the primary lipoic acid ligase of *S*. *aureus*, while LplA2 displays limited activity in vitro in the presence of serum. In *L*. *monocytogenes*, the lipoic acid ligase, LplA1, supports lipoic acid salvage in vivo, but is dispensable in vitro [48]. Based on this information, we reasoned that since a lack of *de novo* lipoic acid biosynthesis did not affect *S*. *aureus* infection of the kidney, perhaps one or both lipoic acid ligases are required to facilitate optimal infection in this organ. To test this hypothesis, we infected 4–6 week old female Swiss Webster mice with the following strains: WT, Δ*lipA*, Δ*lplA1*, Δ*lplA2*, Δ*lplA1*Δ*lplA2*, Δ*lplA1*Δ*lplA2*+*lplA1*, Δ*lplA1*Δ*lplA2*+*lplA2*. At 96 hours post-infection, we isolated kidneys and heart, followed by enumeration of bacterial CFU per organ. In line with our previous results (Fig 3), a Δ*lipA* mutant was significantly compromised for infection of the heart, but not the kidney (Fig 6). Δ*lplA1* and Δ*lplA2* deletion mutants infected both kidneys and hearts to a similar degree as the WT strain. In stark contrast, the Δ*lplA1*Δ*lplA2* double ligase mutant exhibited a 4-log reduction in bacterial CFU in the kidney with modest reductions in the heart (Fig 6). The reduction in bacterial burden was fully complemented by introduction of either *lplA1* or *lplA2* into the double mutant strain. These data indicate that, in contrast to what was seen in vitro, both LplA1 and LplA2 ligases are sufficient to promote infection of the kidney during bloodstream infection. Thus, a pronounced role for LplA2 is identifiable for growth in vivo that is not fully appreciated in vitro (Figs 4 and 5), implying an important function for LplA2 during infection. In the heart, *de novo* lipoic acid biosynthesis plays a critical role in promoting infection, however non-statistically significant reductions in bacterial CFU were observed for Δ*lplA1* and Δ*lplA1*Δ*lplA2* indicating lipoic acid ligase activity also contributes to infection of the heart, albeit less-so than *de novo* biosynthesis.

### Lipoic acid and octanoic acid salvage occurs downstream of the octanoyl transferase activity of LipM, while LipM is required for octanoic acid transfer to GcvH during *de novo* biosynthesis

**Fig 7:**
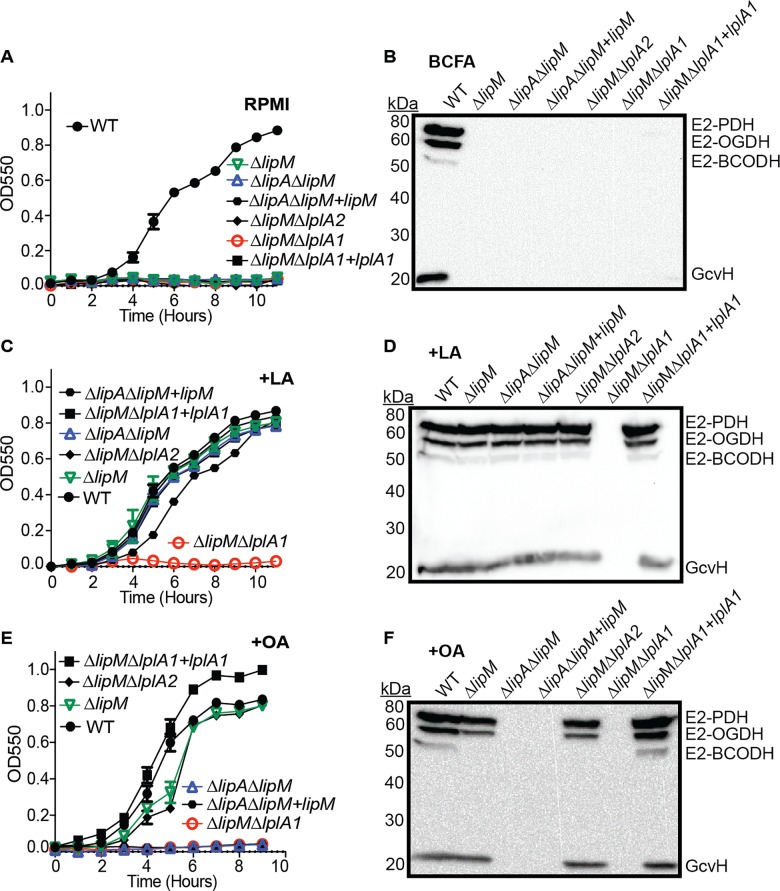
LplA1-dependent salvage of lipoic acid and octanoic acid acts downstream of LipM. (A, C, and E) Growth curves of the indicated strains in (A) RPMI, (C) RPMI + 25nM lipoic acid (LA) or (E) RPMI + 250 μM octanoic acid (OA). (B, D, and F) Whole cell lysates of *S*. *aureus* collected after 9 hours of growth in (B) RPMI + BCFA (2-methyl butyric acid, isovaleric acid, isobutyric acid, and sodium acetate), (D) RPMI + BCFA + 5 μM lipoic acid (LA), or (F) RPMI + BCFA + 175 μM octanoic acid (OA) followed by immunoblotting for lipoic acid-containing proteins. In all growth curves, the mean +/- standard deviation of triplicate measures is shown. In any case where an error bar is not visible, the standard deviation was smaller than the size of the symbol used at that data point.

We have thus far determined that LipA, LipM, and LipL comprise components of the *de novo* lipoic acid biosynthesis pathway and LplA1 and LplA2 constitute enzymes involved in lipoic acid salvage with LplA2 exerting its most notable activity during infection. At this point, we sought to more precisely define the functional role(s) of LipM and more clearly define where LplA1 acts during lipoic acid and octanoic acid salvage in vitro. We reasoned that LipM could conceivably behave exclusively in *de novo* biosynthesis or it could also act as a critical shuttle for octanoyl and lipoyl moieties generated during LplA1-mediated salvage processes. To test these hypotheses, we first generated a set of double mutants in a Δ*lipM* background to determine whether lipoic acid and octanoic acid salvage depends upon *lipM* for subsequent octanoyl and lipoyl transfer. We conducted growth curves and whole cell lysate immunoblots in the presence or absence of exogenous lipoic acid and octanoic acid using the following strains: WT, Δ*lipM*, Δ*lipA*Δ*lipM*, Δ*lipM*Δ*lplA1*, Δ*lipM*Δ*lplA2*, Δ*lipA*Δ*lipM*+*lipM*, and Δ*lipM*Δ*lplA1*+*lplA1* (Fig 7 and S4 Fig). As expected, without supplementation of the medium, no strains were able to grow and protein lipoylation was absent except for WT *S*. *aureus* (Fig 7A and 7B). When lipoic acid was supplemented, all strains except Δ*lipM*Δ*lplA1* were able to grow and lipoylate each of the four metabolic enzyme E2 and H components (Fig 7C and 7D). When octanoic acid was supplemented, all mutant strains lacking *lipA* (Δ*lipA*Δ*lipM*, Δ*lipA*Δ*lipM*+*lipM*), as well as the Δ*lipM*Δ*lplA1* double mutant, were unable to grow or lipoylate proteins (Fig 7E and 7F). These data imply that LplA1 activity does not depend on LipM and that the function of LipM is exclusive to *de novo* lipoic acid biosynthesis.

**Fig 8:**
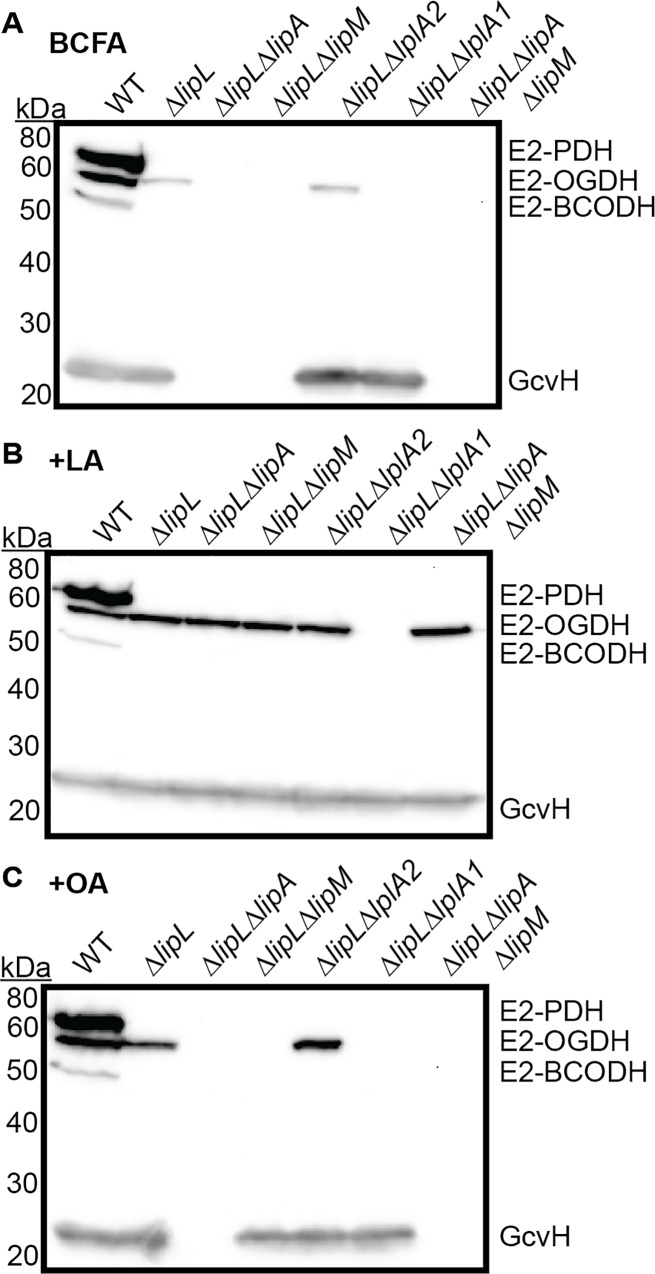
LipM exclusively participates in *de novo* lipoic acid biosynthesis, while LplA1 can ligate free lipoic acid onto OGDH and lipoic acid or octanoic acid onto GcvH. (A-C) Whole cell lysates of *S*. *aureus* collected after 9 hours of growth in (A) RPMI + BCFA (2-methyl butyric acid, isovaleric acid, isobutyric acid, and sodium acetate), (B) RPMI + BCFA + 5 μM lipoic acid (LA), or (C) RPMI + BCFA + 175 μM octanoic acid (OA) followed by immunoblotting for lipoic acid-containing proteins.

We next sought to determine the order of protein lipoylation as dictated by the *de novo* biosynthesis or lipoic acid salvage pathways. Evidence from studies in *B*. *subtilis* indicates that lipoylation of the E2 enzyme subunits depends on prior lipoylation of GcvH [29, 40, 41]. Thus far, we know that LipL contributes, at minimum, to the transfer of lipoyl groups to E2-PDH and E2-BCODH (Fig 2). To decipher the precise order of lipoyl transfer in *S*. *aureus* we generated the following strains in a Δ*lipL* mutant background: Δ*lipL*Δ*lipA*, Δ*lipL*Δ*lipM*, Δ*lipL*Δ*lplA1*, Δ*lipL*Δ*lplA2*, and Δ*lipL*Δ*lipA*Δ*lipM*. Growth curves were not conducted because we previously determined that *lipL* is required for *S*. *aureus* growth irrespective of lipoic acid and octanoic acid supplementation (Fig 2). However, a Δ*lipL* mutant and all mutant derivatives still grow in BCFA medium (S3A and S3E Fig), therefore we exclusively assessed lipoylation profiles by immunoblot (Fig 8 and S7 Fig). When grown in BCFA medium without lipoic acid or octanoic acid, a Δ*lipL* mutant maintained the capacity to lipoylate GcvH and E2-OGDH (Fig 8A). A Δ*lipL*Δ*lipA* double mutant was unable to lipoylate proteins due to the lack of lipoic acid synthetase activity. In addition, the Δ*lipL*Δ*lipM* double mutant was unable to lipoylate any proteins, validating the critical role for LipM in initiating *de novo* biosynthesis by providing octanoyl-protein substrates for LipA. Interestingly, the Δ*lipL*Δ*lplA1* double mutant no longer lipoylated E2-OGDH, implying that *de novo* biosynthesis occurs through initial lipoylation of GcvH after octanoyl transfer by LipM and subsequent lipoic acid synthesis by LipA (Fig 8A). When lipoic acid was supplemented in the medium all mutants had lipoylation patterns that resembled a Δ*lipL* deletion mutant except for Δ*lipL*Δ*lplA1*, which lacks lipoylation on E2-OGDH, confirming a requirement for LplA1 to lipoylate E2-OGDH under these conditions (Fig 8B). After octanoic acid supplementation, strains harboring a *lipA* mutation were unable to lipoylate proteins, whereas both Δ*lipL*Δ*lipM*, and Δ*lipL*Δ*lplA1* were only able to lipoylate GcvH (Fig 8C). Together these data suggest that: (1) the LplA1 ligase is able to use both octanoic acid and lipoic acid as substrates. LplA1 can lipoylate E2-OGDH directly, and does not depend on a committed step through GcvH to do so. In contrast, LplA1 salvage of octanoic acid occurs exclusively through transfer to GcvH; (2) LipM is the only octanoyl transferase involved in *de novo* biosynthesis, where it facilitates the transfer of an octanoyl moiety to GcvH, and is the substrate of LipA; (3) LipL is likely to catalyze lipoyl transfer from GcvH to all three E2 subunits (PDH, OGDH, and BCODH), however dependency on LipL for the transfer of lipoyl moieties to E2-OGDH can be compensated by the activity of LplA1; and (4) a functional role for the LplA2 ligase under the conditions tested cannot be determined.

### Lipoic acid is required to support metabolic programs needed for *S*. *aureus* invasive disease

**Fig 9:**
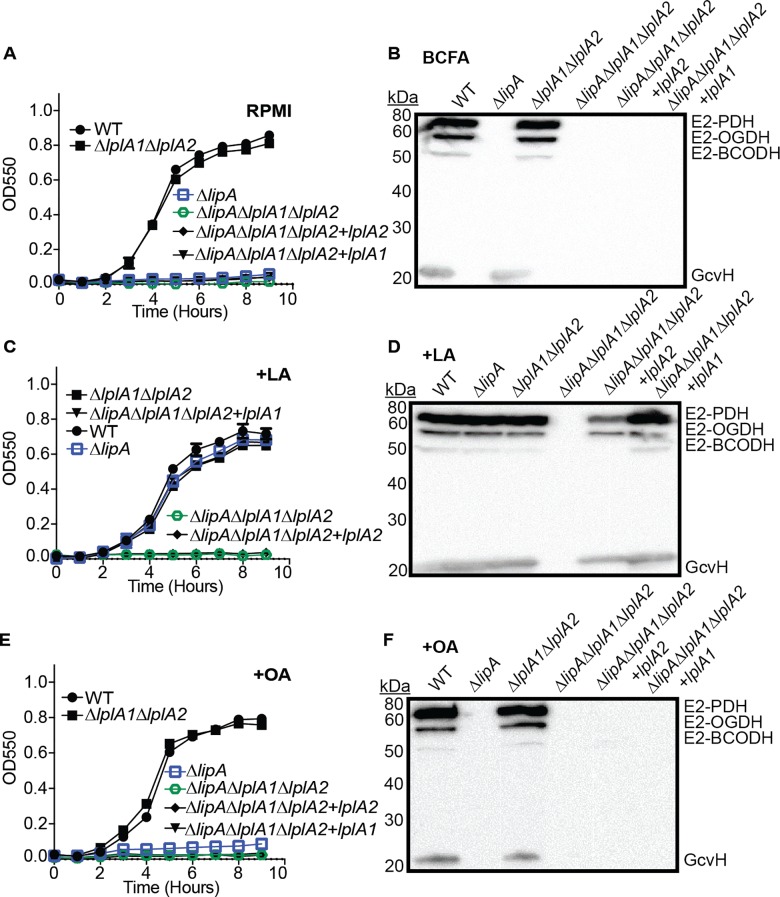
When constitutively expressed, LplA2 can facilitate lipoylation of E2 subunits and GcvH in vitro. (A, C, and E) Growth curves of the indicated strains in (A) RPMI, (C) RPMI + 25 nM lipoic acid (LA) or (E) RPMI + 250 μM octanoic acid (OA). (B, D, and F) Whole cell lysates of *S*. *aureus* collected after 9 hours of growth in (B) RPMI + BCFA (2-methyl butyric acid, isovaleric acid, isobutyric acid, and sodium acetate), (D) RPMI + BCFA + 5 μM lipoic acid (LA), or (F) RPMI + BCFA + 175 μM octanoic acid (OA) followed by immunoblotting for lipoic acid-containing proteins. In all growth curves, the mean +/- standard deviation of triplicate measures is shown. In any case where an error bar is not visible, the standard deviation was smaller than the size of the symbol used at that data point.

Since we identified critical roles for lipoic acid biosynthesis and salvage in promoting bacterial viability in distinct tissue sites we hypothesized that elimination of both salvage and *de novo* biosynthesis of lipoic acid could conceivably have detrimental consequences for the bacterium during infection. However, given the complexity of *S*. *aureus* metabolic programs and nutrient acquisition mechanisms, we wondered whether *S*. *aureus* might be able to shift metabolic programs in vivo to promote survival when faced with lipoic acid starvation. To test this, we used the previously described Δ*lipA*Δ*lplA1*Δ*lplA2* triple mutant and two complementation strains: Δ*lipA*Δ*lplA1*Δ*lplA2*+*lplA1* and Δ*lipA*Δ*lplA1*Δ*lplA2*+*lplA2*. We first assessed the growth and lipoylation efficiency of the Δ*lipA*Δ*lplA1*Δ*lplA2* mutant in vitro (Fig 9 and S7 Fig). Indeed, the triple mutant was unable to replicate in RPMI, RPMI+LA, or RPMI+OA and was unable to lipoylate proteins in BCFA, BCFA+LA, and BCFA+OA (Fig 9A–9F). In contrast, the Δ*lipA*Δ*lplA1*Δ*lplA2*+*lplA1* and Δ*lipA*Δ*lplA1*Δ*lplA2*+*lplA2* ligase complementation strains exhibited key phenotypic differences from one another after lipoic acid supplementation (Fig 9C and 9D). Δ*lipA*Δ*lplA1*Δ*lplA2*+*lplA1* was sufficient to promote growth and lipoylation of all E2 and H subunits (Fig 9B). In contrast, the Δ*lipA*Δ*lplA1*Δ*lplA2*+*lplA2* complementation strain was unable to restore growth in the presence of 25 nM lipoic acid, however, a modest degree of lipoylation was observed on E2-PDH, E2-OGDH, and GcvH, but not E2-BCODH when lipoic acid was in excess (5 μM). Intriguingly, this is the first time we witnessed LplA2 dependent lipoylation of E2-OGDH, and GcvH in vitro suggesting gene expression levels and lipoic acid abundance may facilitate lipoylation by LplA2 in broth culture.

**Fig 10:**
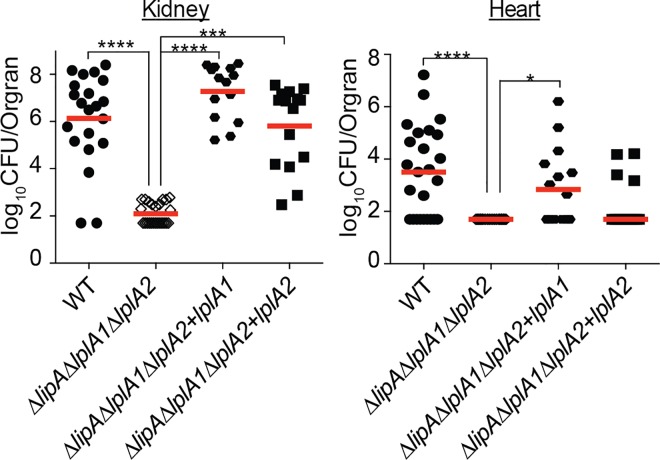
*aureus* requires lipoic acid to establish bloodstream infection and either LplA1 or LplA2 is sufficient to restore infectious burden to the kidney. ***S*.** Bacterial burden in kidneys and hearts of mice 96 hours after infection via the bloodstream with 1.0 x 10^7^ CFU WT, Δ*lipA*Δ*lplA1*Δ*lplA2*, Δ*lipA*Δ*lplA1*Δ*lplA2+lplA1*, and Δ*lipA*Δ*lplA1*Δ*lplA2+lplA2* strains. log_10_CFU per organ is displayed for each infected mouse along with the median as a measure of central tendency. Animal numbers are as follows: WT, *N* = 21; Δ*lipA*Δ*lplA1*Δ*lplA2*, *N* = 20; Δ*lipA*Δ*lplA1*Δ*lplA2+lplA1*, *N* = 14; Δ*lipA*Δ*lplA1*Δ*lplA2+lplA2*, *N* = 15. Statistics were calculated using a nonparametric 1-way ANOVA with Kruskal-Wallis multiple comparisons post-test to evaluate statistical significance between groups. Statistically significant differences are as indicated in the figure. ****, *P*<0.0001; ***, *P*<0.001; and *, *P*<0.05.

With confirmation that the Δ*lipA*Δ*lplA1*Δ*lplA2* triple mutant was defective for both *de novo* biosynthesis and lipoic acid salvage, we then proceeded to infect mice with the same strains and evaluated bacterial burden in kidneys and hearts 96 hours post-infection (Fig 10). Δ*lipA*Δ*lplA1*Δ*lplA2* was nearly avirulent with most kidneys and hearts at or near the limit of detection for recoverable CFU. Both complemented strains restored infection in the kidney to WT or near WT levels for Δ*lipA*Δ*lplA1*Δ*lplA2*+*lplA1* and Δ*lipA*Δ*lplA1*Δ*lplA2*+*lplA2* respectively, consistent with their perceived differences in lipoylation efficiency in vitro (Fig 9). In contrast, levels of Δ*lipA*Δ*lplA1*Δ*lplA2*+*lplA1* and Δ*lipA*Δ*lplA1*Δ*lplA2*+*lplA2* in infected hearts were only partially restorative, in support of our previous findings that suggest *de novo* biosynthesis plays a dominant role in infection of the heart. Together, these data indicate that the composite functions of lipoic acid biosynthesis and salvage are required to establish infection and highlight the fact that compensatory metabolic strategies are not engaged in vivo to offset the lack of lipoic acid incorporation. Furthermore, lipoic acid ligases have a high capacity to acquire lipoic acid in permissive tissues, where their activity alone can permit bacterial survival even when *de novo* biosynthesis is lacking.

## Discussion

In this study we used a genetic approach to determine the mechanisms of lipoic acid biosynthesis and salvage in the Gram-positive pathogen *S*. *aureus*. We then used this information to examine how lipoic acid acquisition strategies facilitate pathogenic outcomes in a murine model of sepsis. Our findings highlight the unique roles of both lipoic acid biosynthesis and salvage pathways in dictating niche-specific infection outcomes in vivo. Further, our data highlight the critical importance of trace nutrient acquisition schemes for pathogenesis when in nutrient-limited environments.

### Lipoic acid *de novo* biosynthesis and salvage pathways converge to yield a complex utilization scheme in *S*. *aureus*

**Fig 11:**
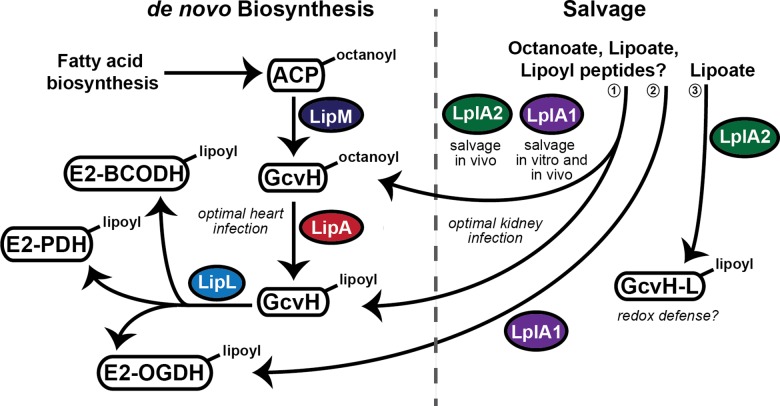
Model of lipoic acid biosynthesis and salvage pathways in *S*. *aureus*. *S*. *aureus* is capable of both *de novo* biosynthesis of lipoic acid and salvage of the cofactor from environmental sources. LipM, LipA, and LipL comprise the three main enzymes involved in *de novo* biosynthesis, whereby octanoic acid, bound to an acyl carrier protein (ACP) from fatty acid biosynthesis is transferred, by LipM, to the H protein of the Glycine cleavage system (GcvH). Octanoyl-GcvH acts as a substrate for LipA, which generates lipoyl-GcvH. LipL then transfers the lipoyl group of GcvH to the three lipoyl-containing E2 subunits of the pyruvate dehydrogenase (PDH), 2-oxoglutarate dehydrogenase (OGDH), and branched chain 2-oxoacid dehydrogenase (BCODH) complexes encoded by *S*. *aureus*. *de novo* biosynthesis of lipoic acid is critical for systemic infection and plays a major role in promoting infection of the heart, but not the kidney. Salvage of lipoic acid occurs through the action of two ligases, LplA1 and LplA2. (1) LplA1 functions in vitro and in vivo and facilitates attachment of free lipoic acid/octanoic acid to GcvH. LplA2 is dispensable in vitro, but is functional in vivo where it acts in concert with LplA1 to promote bacterial survival in the kidney. (2) LplA1 is also able to directly ligate free lipoic acid onto the E2 subunit of the OGDH complex in vitro. (3) LplA2 has high affinity for the GcvH-like protein GcvH-L, where its lipoylation promotes subsequent ADP-ribosylation of GcvH-L. The lipoylation and ADP-ribosylation of GcvH-L is hypothesized to facilitate redox defense during infection.

Based upon our findings and those of Rack *et al*, we have proposed a model for lipoic acid acquisition in *S*. *aureus* (Fig 11) [42]. In this model, *S*. *aureus* engages in both *de novo* biosynthesis of lipoic acid as well as lipoic acid salvage. In many ways, these two pathways resemble the *de novo* biosynthesis and salvage mechanisms used by *B*. *subtilis* and *L*. *monocytogenes* respectively, though the *S*. *aureus* pathway bears a number of novel features discussed below. Bacterial growth and lipoylation profile analyses of lipoic acid biosynthesis gene deletion mutants support a model of *de novo* biosynthesis that includes three enzymes: LipM, LipA, and LipL. Like *B*. *subtilis*, we propose that *S*. *aureus* LipM is an octanoyl transferase, required for the transfer of octanoic acid to the H protein of the glycine cleavage system; LipA a lipoic acid synthetase responsible for the conversion of octanoyl-GcvH to lipoyl-GcvH; and LipL, an amidostransferase that transfers the lipoyl moiety from lipoyl-GcvH to E2-PDH, E2-OGDH, and E2-BCODH. When a Δ*lipL* mutant is grown in BCFA medium lacking lipoic acid or octanoic acid, GcvH is lipoylated to the same extent as WT, while E2-PDH and E2-BCODH are no longer lipoylated. This finding suggests LipL, at minimum, transfers the lipoyl group from GcvH to these two E2 subunits. In contrast, E2-OGDH shows a limited degree of lipoylation when a Δ*lipL* mutant is grown in BCFA medium. We determined that the low levels of lipoyl-E2-OGDH are a result of the activity of LplA1, though this appears to be secondary to its primary function as a lipoate ligase involved in salvage (see below). Under standard growth conditions we propose lipoyl-GcvH is the lipoic acid source used by LipL during lipoyl transfer to all three E2 subunits, especially under conditions where lipoic acid salvage is dispensable. However, when salvage is permitted by lipoic acid supplementation in the culture medium, all E2 subunits can be lipoylated in the absence of GcvH (S5 Fig). This indicates there is an absolute requirement for lipoyl-GcvH in *de novo* biosynthesis that is only bypassed through the activity of salvage ligases when exogenous lipoic acid is present. As it stands, our model depicted in Fig 11 has not yet been validated at the biochemical level therefore we are actively pursuing biochemical studies that will provide additional clarity into the function and specificity of both LipL and LplA1 for all three E2 subunits. Furthermore, while our proposed functionalities for LipL closely resemble that of *B*. *subtilis* LipL, the co-translational coupling associated with the *pta-lipL* operon that prevented successful complementation with *lipL* alone (S2 Fig) implies functions of Pta could conceivably contribute to some of the growth and lipoylation phenotypes observed with a Δ*lipL* mutant. However, because our data coincides with ascribed functions for LipL in *B*. *subtilis*, we suggest a mechanism of *de novo* lipoic acid biosynthesis that comprises transfer of an octanoyl group to GcvH by LipM; generation of lipoyl-GcvH by LipA; and transfer of lipoyl groups to all three *S*. *aureus* metabolic E2 subunits by LipL.

While *de novo* biosynthesis of lipoic acid bears a striking resemblance to that of *B*. *subtilis*, the lipoic acid salvage strategies of *S*. *aureus* diverge [29]. *S*. *aureus* contains two predicted lipoic acid salvage enzymes, LplA1 and LplA2, with moderate amino acid sequence similarity to LplJ of *B*. *subtilis* and LplA1 and LplA2 of *L*. *monocytogenes*. As mentioned in our results, LipM and LipL are annotated as lipoic acid ligases in most *S*. *aureus* genome databases, leading to their designation as LplA3 and LplA4 in the literature [42]. Our bioinformatics analyses indicate this designation is likely due to their association with protein family, PFAM03099, which can exhibit a wide variety of activities associated with both *de novo* biosynthesis and lipoic acid salvage. However, our studies and those of Rack *et al*, indicate that LplA1 and LplA2 are the only lipoic acid ligases in *S*. *aureus*, while the designators LplA3 and LplA4 correspond to LipM and LipL respectively [42]. Rack *et al* have shown that both LplA1 and LplA2 are capable of lipoylating both GcvH and GcvH-L in vitro, although LplA2 has greater affinity for GcvH-L than LplA1 [42]. The authors reason that this affinity is predicted by the fact that *lplA2* is present in an operon with *gcvH-L* (discussed below). Our data indicate that LplA1 is required for salvage of both lipoic acid and octanoic acid in vitro. In contrast, no detectable role in lipoic acid salvage was identified for LplA2 under standard growth conditions. However, we found that when a Δ*lipA*Δ*lplA1* double mutant is grown in the presence of murine bone marrow derived macrophages or in medium supplemented with 20% FBS, in vitro growth is almost completely restored and limited lipoylation of E2-PDH is detected. In contrast, a Δ*lipA*Δ*lplA2* double mutant is unable to replicate in FBS unless exogenous lipoic acid is also supplemented into the base medium. We suspect that in the presence of FBS the expression of *lplA2* is induced to a sufficient degree such that limited functionality is observed in broth culture. These findings imply a role for LplA2 in lipoylation when its expression is sufficiently high, or when in the presence of alternative lipoyl substrates that might be present in FBS (lipoyl peptides, lipoamide, etc.). Implicit in the suggestion that LplA2 may use lipoyl peptides or lipoamide is the requirement for lipoamidase activity to liberate free lipoic acid from its peptide bound form. In light of our findings that demonstrate LplA2, but not LplA1, dependency for growth in FBS we hypothesize that *S*. *aureus* either possesses a dedicated lipoamidase or that LplA2 may have dual lipoamidase/ligase activity. We are actively pursuing studies to test these possibilities. Nevertheless, the most striking evidence for LplA2 ligase functionality is that it acts in concert with LplA1 to promote kidney infection during murine sepsis. Based on our composite data, we hypothesize that LplA2 is either exposed to its optimal substrates or its gene expression is upregulated in vivo such that the activity of the enzyme is fully realized. Evidence in support of these hypotheses stems directly from our macrophage and serum supplementation studies described above, as well as our *lplA2* complementation studies (Figs 5 and 9). While re-introduction of *lplA2* into a Δ*lipA*Δ*lplA1*Δ*lplA2* triple mutant does not restore bacterial growth in the presence of low levels of lipoic acid, it does permit partial lipoylation when lipoic acid is provided in low micromolar concentrations. In this complementation construct *lplA2* gene expression is driven by a constitutive promoter, *P*_*HELP*_, therefore the modest lipoylation seen may be a direct consequence of uncoupling LplA2 function from restrictions imposed by gene expression levels. We propose that LplA2 ligase activity potentially permits two outcomes in vivo: (i) it participates in lipoic acid salvage to the extent that it can compensate for a lack of LplA1 (Figs 6 and 10); and (ii) it lipoylates GcvH-L where it is hypothesized to facilitate redox defense, although this function remains to be fully evaluated [42]. In contrast, LplA1 acts as a ligase in vitro and in vivo, and uses either lipoic acid or octanoic acid as a substrate. Interestingly, when *de novo* biosynthesis of lipoic acid is blocked, LplA1 is able to lipoylate GcvH and E2-OGDH directly, suggesting an affinity for these two subunits that does not exist for E2-PDH and E2-BCODH. Detailed biochemical studies will clarify the functional activities of LplA1 and LplA2.

### Novel gene arrangements for *lplA2* and *lipL*

A recent study on bacterial sirtuins identified a conserved locus in a number of bacterial pathogens containing core genes encoding a sirtuin (SirTM), its linked macrodomain protein, GcvH-L, and LplA2 [42]. Interestingly, the function of bacterial sirtuins is often linked to maintenance of metabolic homeostasis [65]. Therefore, it is tempting to surmise that the genetic linkage of *lplA2* to *sirTM* imparts a novel metabolic regulatory program on *S*. *aureus* that relies on protein lipoylation. Indeed, Rack *et al* found that lipoylation of GcvH-L by LplA2 promotes subsequent ADP-ribosylation by the operon-encoded SirTM [42]. ADP-ribosylation was found to be reversible, and is mediated by the function of the macrodomain-containing protein, MACRO. Furthermore, this study suggested that novel crosstalk between lipoylation and ADP-ribosylation could conceivably promote redox defense during infection [42]. This hypothesis has not yet been tested for *S*. *aureus*. Nonetheless, the possibility that redox defense is enhanced by LplA2-mediated lipoylation of GcvH-L could conceivably provide a partial explanation for the reduced virulence seen upon infection with a Δ*lplA1*Δ*lplA2* double mutant in the sepsis model (Fig 6). However, it is also possible that lipoyl-GcvH-L serves as a secondary substrate for LipL, thereby providing an additional source of lipoic acid in scenarios where salvage demands and redox stress is high, such as during infection. Although LplA2 has reduced lipoylation efficiency for GcvH, whether or not LplA2 effectively facilitates lipoylation of E2 subunits of *S*. *aureus* under normal conditions remains to be fully elucidated [42]. Our data, which indicates LplA2 facilitates lipoylation of E2-PDH in the presence of serum as well as E2-PDH and E2-OGDH when constitutively expressed, indicates that, at minimum, LplA2 is capable of facilitating lipoylation of E2s when conditions are permissive. We hypothesize that LplA2-dependent lipoylation is also realized in vivo in light of our data that shows LplA1 and LplA2 can compensate for one another during infection of the kidney (Fig 6).

In addition to operon-encoded LplA2, the gene encoding LipL is also arranged in an operon in *S*. *aureus*. *lipL* is genetically linked to *pta*, which encodes the phosphotransacetylase, Pta. Pta is one of two enzymes in the phosphotransacetylase-acetate kinase (Pta-AckA) pathway, involved in the generation of acetate from Acetyl-CoA [52, 53]. We believe this to be the first reported case of such a linkage between genes encoding a lipoyl transferase and this fermentative enzyme. During the construction of the Δ*lipL+lipL* complementation strain used in this study we found that full complementation of the *lipL* mutation was only achieved after introducing the entire *pta-lipL* locus. The *pta* and *lipL* genes are separated from one another by two nucleotides and appear to share the same ribosome binding site 5 base pairs upstream of the *pta* translation start codon, therefore we believe it is likely that these genes are co-translated. Our complementation analyses suggest this genetic linkage is crucial to the optimal activity of LipL, since complementation with *pta* or *lipL* alone is insufficient to restore WT lipoylation patterns to a Δ*lipL* mutant (S2 Fig). Thus, this genetic arrangement is suggestive of an intimate relationship between acetate metabolism and LipL activity. Previously, LipL *of L*. *monocytogenes was* found to co-precipitate with the PDH complex, thereby linking its activity to E2-subunit of PDH [49]. The Pta-Ack metabolic pathway depends on substrates generated by the PDH complex (Acetyl-CoA) [52]. Therefore, linkage of LipL to acetate metabolism machinery in *S*. *aureus* may imply additional regulatory functions that dictate the entry of Acetyl-CoA into fermentative (Pta-Ack) or oxidative (TCA cycle) metabolic pathways, although this remains to be tested.

### Use of *de novo* biosynthesis and salvage to promote tissue infectivity

Our results demonstrate that *de novo* biosynthesis and salvage of lipoic acid are each critical for pathogenesis at specific infectious sites during systemic infection with *S*. *aureus*. We found that the ability of *S*. *aureus* to infect the hearts of mice requires *de novo* biosynthesis over that of salvage. In contrast, in the kidney, either LplA1 or LplA2 is sufficient for infectivity. These findings provide insight into potential reasons behind the ability of *S*. *aureus* to infect diverse tissues including skin, bone, and virtually any internal organ. Each of these sites of infection presents *S*. *aureus* with unique nutritional restrictions and environmental conditions that demand significant adaptability. Free lipoic acid is rapidly metabolized in the host and remains protein bound under most circumstances, therefore this cofactor is likely to be limiting, although certain organs are believed to contain more protein-bound lipoic acid than others [51, 64]. For example, in rats the amount of protein bound lipoic acid is four times as high in kidneys as it is hearts. Assayed rat kidneys and hearts contain ~4 nmol/g and ~1 nmol/g lipoic acid respectively [51]. As such, maintaining both lipoic acid biosynthesis and salvage pathways would provide maximal adaptability during infection by allowing *S*. *aureus* to acquire the nutrient at sites with more or less protein-bound lipoic acid. It is interesting that infection of the kidney is highly dependent on lipoic acid salvage. We surmise that either the kidney microenvironment of mice, like rats, contains sufficient free and protein-bound lipoic acid to allow preference for salvage pathways in this site, or other gene regulatory events impose restrictions on *de novo* biosynthesis such that salvage is required. In either case, the strict dependency on lipoic acid salvage in one organ versus another is remarkable. It remains to be determined what roles lipoic acid biosynthesis and salvage play in other models of infection or in other tissues.

### Conclusions

Our current study has demonstrated that the mechanisms of lipoic acid biosynthesis and salvage in *S*. *aureus* confer unique adaptive traits that facilitate *S*. *aureus* survival in vitro and at diverse tissue sites during infection. Clearly, there is much to be learned about these pathways in *S*. *aureus*, as well as other pathogenic bacteria. As previously alluded to in earlier studies by Martin *et al*, enzymes in this system with limited homology to those in eukaryotes, such as LipL, are likely to be valuable candidates for therapeutic design due to their crippling effects on bacterial replication [41].

## Materials and Methods

### Bacterial strains and culture conditions

All bacterial strains used in this study are listed in Table 2. *S*. *aureus* pulse field gel electrophoresis type USA300 isolate LAC, cured of its plasmids (wild type–AH1263), was used as the parental strain for all genetic manipulations [66]. *S*. *aureus* NCTC8325 derivative RN4220, *S*. *aureus* RN9011, *E*. *coli* DH5α and DC10B were used as host strains for propagation of recombinant plasmids and plasmid integration.

**Table 2:** List of strains used in this study.

Strain	Genotype	Designation	Source/ Reference
USA300 LAC	*S*. *aureus* USA300 Strain LAC (AH-1264). Plasmid cured.	LAC (WT)	[66]
DH5α	*E*. *coli* strain for recombinant pIMAY and pJC plasmids		
RN4220	Restriction deficient *S*. *aureus* for plasmid passage	RN4220	[67]
RN9011	RN4220 + pRN7203 expressing SaPI integrase	RN9011	[68]
FA-S831	AH-LAC with an in-frame deletion of *lipA*	Δ*lipA*	This work
FA-S842	AH-LAC with an in-frame deletion of *lipM*	Δ*lipM*	This work
FA-S841	AH-LAC with an in-frame deletion of *lplA1*	Δ*lplA1*	This work
FA-S837	AH-LAC with an in-frame deletion of *lplA2*	Δ*lplA2*	This work
FA-S1176	AH-LAC with an in-frame deletion of *lipL*	Δ*lipL*	This work
FA-S1182	AH-LAC with in-frame deletions of *lipA* and *lipM*	Δ*lipA*Δ*lipM*	This work
FA-S1249	AH-LAC with in-frame deletions of *lipA* and *lplA1*	Δ*lipA*Δ*lplA1*	This work
FA-S1180	AH-LAC with in-frame deletions of *lipA* and *lplA2*	Δ*lipA*Δ*lplA2*	This work
FA-S977	AH-LAC with in-frame deletion of *lipA* and Δ*lipL*:*kan*	Δ*lipA*Δ*lipL*	This work
FA-S912	AH-LAC with in-frame deletions of *lplA1* and *lplA2*	Δ*lplA1*Δ*lplA2*	This work
FA-S1251	AH-LAC with in-frame deletions of *lipM* and *lplA1*	Δ*lipM*Δ*lplA1*	This work
FA-S957	AH-LAC with in-frame deletion of *lipM* and transposon insertion in *lplA2* transduced from NE266 (*lplA2*:*erm*)	Δ*lipM*Δ*lplA2*	This work
FA-S994	AH-LAC with in-frame deletion of *lipM* and Δ*lipL*:*kan*	Δ*lipM*Δ*lipL*	This work
FA-S1210	AH-LAC with in-frame deletions of *lipL* and *lplA1*	Δ*lipL*Δ*lplA1*	This work
FA-S998	AH-LAC with in-frame deletions of *lplA2* and Δ*lipL*:*kan*	Δ*lipL*Δ*lplA2*	This work
FA-S1178	AH-LAC with in-frame deletions of *lipA*, *lplA1*, and *lplA2*	Δ*lipA*Δ*lplA1*Δ*lplA2*	This work
FA-S992	AH-LAC with in-frame deletion of *lipA*, Δ*lipL*:*kan*, and transposon insertion in *lipM* transduced from NE1334	Δ*lipL*Δ*lipA*Δ*lipM*	This work
FA-S1038	AH-LAC with in-frame deletion of *gcvH*	Δ*gcvH*	This work
FA-S1041	AH-LAC with in-frame deletion of *E2-PDH*	Δ*E2-PDH*	This work
FA-S1042	AH-LAC with in-frame deletion of *E2-OGDH*	Δ*E2-OGDH*	This work
FA-S877	FA-S831 complemented with pJC1112-*lipA*	Δ*lipA+lipA*	This work
FA-S1119	FA-S842 complemented with pJC1111-*lipM*	Δ*lipM+lipM*	This work
FA-S1190	FA-S1176 complemented with pJC1111-*lipL*	Δ*lipL+lipL*	This work
FA-S1258	FA-S1176 complemented with pJC1111-*pta*	Δ*lipL+pta*	This work
FA-S1257	FA-S1176 complemented with pJC1111-*pta-lipL*	Δ*lipL+pta-lipL*	This work
FA-S1206	FA-S912 complemented with pJC1111-*lplA1*	Δ*lplA1*Δ*lplA2*+*lplA1*	This work
FA-S1208	FA-S912 complemented with pJC1111-*lplA2*	Δ*lplA1*Δ*lplA2*+*lplA2*	This work
FA-S1259	FA-S1249 complemented with pJC1111-*lplA1*	Δ*lipA*Δ*lplA1+lplA1*	This work
FA-S1205	FA-S1180 complemented with pJC1111-*lplA2*	Δ*lipA*Δ*lplA2+lplA2*	This work
FA-S1260	FA-S1251 complemented with pJC1111-*lplA1*	Δ*lipM*Δ*lplA1+lplA1*	This work
FA-S1222	FA-S1182 complemented with pJC1111-*lipM*	Δ*lipA*Δ*lipM+lipM*	This work
FA-S1200	FA-S1178 complemented with pJC1111-*lplA1*	Δ*lipA*Δ*lplA1*Δ*lplA2+lplA1*	This work
FA-S1212	FA-S1178 complemented with pJC1111-*lplA2*	Δ*lipA*Δ*lplA1*Δ*lplA2+lplA2*	This work

All *E*. *coli* strains were grown in Lysogeny Broth (LB) (Amresco) supplemented with antibiotics as indicated below. For in vitro culture of *S*. *aureus* strains, Tryptic Soy Broth (TSB) (Criterion) was used as rich medium, and Roswell Park Memorial Institute 1640 medium (RPMI) (Corning) supplemented with 1% casamino acids (Amresco) was used as a defined medium lacking lipoic acid and octanoic acid. Unless otherwise specified, cultures were incubated at 37°C in a shaking incubator at 200 rpm with tubes held at a 45° angle. When required, LB and TSB media were solidified using 1.5% Agar (Amresco).

Where necessary, media was supplemented with the following antibiotics or chemicals at the following final concentrations: ampicillin (Amp) at 100 μg/mL, erythromycin (Erm) at 3 μg/mL, kanamycin (Kan) at 50 μg/mL, neomycin (Neo) at 50 μg/mL, chloramphenicol (Cam) at 10 μg/mL (Amresco), anhydrous tetracycline (AnTet) (Acròs Organics) at 1 μg/mL, CdCl_2_ (Alfa Aesar) 0.3 mM, sodium citrate (Sigma) 10 mM. Medium used for experiments where the requirement for lipoic acid or octanoic acid had to be bypassed were supplemented with the following short branched-chain carboxylic acids at the indicated concentrations (Sigma): 10.8 mM isobutyric acid, 9.2 mM 2-methylbutyric acid and 9 mM isovaleric acid, and 10 mM sodium acetate (Sigma) [41, 61]. RPMI or TSB medium containing these supplements bypasses the metabolic requirement for lipoylated enzyme complexes involved in TCA cycle and branched chain amino acid catabolism and is referred as +BCFA throughout.

### Molecular genetic techniques

*S*. *aureus* chromosomal DNA was isolated using Wizard Genomic DNA purification kit following the manufacturers protocol with the following modifications (Promega). 2.5 μL of lysostaphin (Ambi Products, NY) stock solution (2 mg/mL in 20 mM sodium acetate, pH 4.5) was added to a 1.2 mL culture of *S*. *aureus* that had first been pelleted by centrifugation and resuspended in 200 μL of TSM buffer (50mM Tris, 0.5M D-Sucrose,10 mM MgCl_2_ pH 7.5), followed by incubation for 15 minutes at 37°C to digest the cell wall. After lysostaphin treatment, the bacteria were centrifuged at maximum speed for 3 minutes in a microcentrifuge and bacterial DNA extracted following the remainder of the manufacturer’s protocol. QIAGEN Mini / Midi plasmid isolation kits were used to extract recombinant plasmids. DNA gel extraction was performed using a QIAGEN QIAquick Gel Extraction kit. Polymerase chain reaction (PCR) was performed in Flexid Mastercycler (eppendorf) using Phusion High-Fidelity DNA Polymerase (New England Biolabs), oligonucleotides from Eurofins and dNTPs from Quanta Biosciences. DNA ligation was performed in eppendorf ThermoMixer C using T4 DNA ligase (New England Biolabs). PCR purification was done using a QIAquick PCR purification kit from QIAGEN. DNA sequencing analysis was performed by Genscript. All restriction endonucleases were purchased from New England Biolabs.

### *E*. *coli* competent cell preparation

A 3 mL culture of *E*. *coli* was grown overnight at 37°C in a shaking incubator. The following day, the strain was subcultured at a 1:55 dilution (2 mL into 110 mL) into fresh LB in a 250 mL flask. Bacteria were incubated at 37°C with shaking for ~2.5 hours until reaching mid-logarithmic phase (OD600 0.3–0.4). Four 50 mL tubes containing 25 mL of the log-phase culture were kept on ice for a period of 10 minutes, after which they were centrifuged at 4,000 rpm for 10 minutes at 4°C. Bacterial cells were harvested after decanting the supernatant and washed twice in 10 mL of filter-sterilized Transformation Buffer 1 (TFB-1) (30 mM KOAc, 100 mM RbCl_2_, 10 mM CaCl_2_, 50 mM MnCl_2_, 15% Glycerol, adjusted at pH 5.8 with 0.2 M Acetic Acid) (Amresco). Cells were incubated on ice for 10 minutes between washes. After a final centrifugation at 4,700 rpm for 5 minutes the bacterial pellet was suspended in 1 mL of filter-sterilized Transformation Buffer 2 (TFB-2) (10 mM MOPS, 10 mM RbCl_2_, 75 mM CaCl_2_, 15% Glycerol, adjusted at pH 6.5 with KOH) (Amresco). Finally, 100μL of the competent cells were immediately aliquoted into 1.5 mL microcentrifuge tubes and stored at -80°C for future use.

### *E*. *coli* transformation

In order to transform chemically competent *E*. *coli*, 2 μL of the ligation product or purified plasmid was incubated with 50 μL competent cells on ice for 20 min. Afterwards, cells were incubated at 42°C for 45 seconds, placed on ice for 2 minutes and then resuspended in 250 μL of SOC medium (0.5% tryptone, 0.5% yeast extract, 0.05% NaCl, 250 mM KCl adjusted to a pH 7.0 using 5M NaOH followed by addition of 20 mL 1M glucose) (Amresco). After heat shock, cells were allowed to recover with shaking at 30°C for 1 hour, after which 100 μL were spread onto LB selection plates containing antibiotic supplements.

### Preparation of *S*. *aureus* electrocompetent cells

*S*. *aureus* competent cells were prepared by inoculating 300 μL of an overnight culture into 30 mL of fresh TSB. Cells were incubated with shaking at 37°C for 3 hours until reaching mid-logarithmic stage followed by centrifugation at 8,000 rpm for 10 minutes at 4°C. The bacterial pellet was washed 2 times in 30 mL ice-cold 10% glycerol. Cells were then suspended in 15 mL 10% glycerol followed by additional centrifugation at 8,000 rpm for 10 minutes. Lastly, cells were suspended in 3 mL 10% glycerol and 200–500 μL aliquots were distributed into microcentrifuge tubes and stored at -80°C. Strains that harbored antibiotic resistance cassettes or showed impaired growth due to deficiencies in lipoic acid biosynthesis were supplemented with antibiotics and/or BCFA medium respectively prior to growth.

### *S*. *aureus* transformation via electroporation

Transformation was performed by incubating a mixture of 50 μL thawed *S*. *aureus* competent cells with 10 μL of purified plasmid (~1 μg) at room temperature for 30 minutes. Cells were transferred to a 2 mm electroporation cuvette (VWR) and pulsed at 1,800 V, 10 μF, and 600Ω in a GenePulser Xcell BIORAD electroporator. Cells were allowed to recover in TSB or TSB+BCFA as needed by incubating for 1 hour and 30 min at 30°C. Afterwards, 100 μL were spread onto TSA plates supplemented with the appropriate antibiotics and incubated at 30°C for 24–48 hours.

### Construction of mutagenesis vectors

Two fragments corresponding to ~500 nucleotides upstream of the start codon (ATG) and ~500 nucleotides downstream from the stop codon (TAA) of the target gene of interest were amplified using oligonucleotide pairs shown in Table 3. Briefly, each upstream fragment was PCR amplified from wild type *S*. *aureus* genomic DNA using oligonucleotide #1 (0829SOE1-Kpn; 1494SOE1-Kpn; 0930SOE1-Kpn; 0930SOE1-Kpn; 0328SOE1-Kpn; 0571SOE1-Kpn; 0791SOE1-Kpn; 0995SOE1-Kpn; 1305SOE1-Kpn and 1464SOE1-Kpn) and oligonucleotide #2 (0829SOE2-Kas; 1494SOE2-Kas; 0571SOE2-Kas; 0328SOE2-Kas; 0930SOE2-Kas; 0995SOE2-Kas; 1305SOE2-Kas and 1464SOE2-Kas). Downstream fragments were generated by PCR amplification using oligonucleotide #3 (0829SOE3-Kas;1494SOE3-Kas; 0571SOE3-Kas; 0328SOE3-Kas; 0930SOE3-Kas; 0791SOE3-Kas; 0995SOE3-Kas; 1305SOE3-Kas and 1464SOE3-Kas) and oligonucleotide #4 (0829SOE4-Sac; 1494SOE4-Sac; 0571SOE4-Sac; 0328SOE4-Sac; 0930SOE4-Sac; 0995SOE4-Sac; 1305SOE4-Sac; 1464SOE4-Sac). Both upstream and downstream amplicons were purified and used as templates in a splicing by overlap extension (SOEing) PCR reaction to obtain the final amplicon required for mutagenesis. Each amplicon of ~1000 nucleotides was subcloned into pIMAY using KpnI and SacI restriction endonucleases.

**Table 3:** List of oligonucleotides used in this study.

Name	Sequence
0829SOE1-Kpn	CCC-GGTACC(KpnI)-GCACAATGTGCCATCATCAA
0829SOE2-Kas	CCTTATTAATGGTTAAATATTAACT-GGCGCC(KasI)-CGCCATAACAACACATACCC
0829SOE3-Kas	GGGTATGTGTTGTTATGGCG-GGCGCC(KasI)-AGTTAATATTTAACCATTAATAAGG
0829SOE4-Sac	CCC-GAGCTC(SacI)-ATTAATGTTCAGTATCTTGAATG
0930SOE1-Kpn	CCC-GGTACC(KpnI)-GCATTATACCTGTATAAATAC
0930SOE2-Kas	ATAGTCCTTTAATCGTTTATGA-GGCGCC(KasI)-TTTCATTACAATCTCTCCCTT
0930SOE3-Kas	AAGGGAGAGATTGTAATGAAA-GGCGCC(KasI)-TCATAAACGATTAAAGGACTAT
0930SOE4-Sac	CCC-GAGCTC(SacI)-ATAAGCAAAACCTCGCTTTAT
0328SOE1-Kpn	CCC-GGTACC(Kpn1)-TATTTCATATACAAGGGGAGTATA
0328SOE2-Kas	CATACATAAAATAACAATATTAACT-GGCGCC(KasI)-GTACATCTTCTGTCTCTCCA
0328SOE3-Kas	TGGAGAGACAGAAGATGTAC-GGCGCC(KasI)-AGTTAATATTGTTATTTTATGTATG
0328SOE4-Sac	CCC-GAGCTC(SacI)-CTTGTGATAAATCCGCTTCG
1494SOE1-Kpn	CCC-GGTACC(KpnI)-TTATAGCCGCCTTTTAACATA
1494SOE2-Kas	CATTTTTTATTCTAAAAACTACTT-GGCGCC(KasI)-AGTCATATTCAAGAACTCCTA
1494SOE3-Kas	TAGGAGTTCTTGAATATGACT-GGCGCC(KasI)-AAGTAGTTTTTAGAATAAAAAATG
1494SOE4-Sac	CCC-GAGCTC(SacI)-ATATTCAAAGTGCTCACACTT
0571SOE1-Kpn	CCC-GGTACC(KpnI)-AAGTGCAAAATCAGCATTAAG
0571SOE2-Kas	GGCTGTTAAATATTTAACTATTG-GGCGCC(KasI)-TTGCATAGGTTCTAATCCAG
0571SOE3-Kas	CTGGATTAGAACCTATGCAA-GGCGCC(KasI)-CAATAGTTAAATATTTAACAGCC
0571SOE4-Sac	CCC-GAGCTC(SacI)-GTTGCATACGCATTCTCGT
0791SOE1-Kpn	CCC-GGTACC(KpnI)-AGGTTGCAGTCGTATGATTA
0791SOE2-Kas	ATTAAGGAGTTACACGGTGA-GGCGCC(KasI)-GAGAATCCCCTCCTAATTAA
0791SOE3-Kas	TTAATTAGGAGGGGATTCTC-GGCGCC(KasI)-TCACCGTGTAACTCCTTAAT
0791SOE4-Sac	CCC-GAGCTC(SacI)-CAGACATTCCATACATTTGATC
0995SOE1-Kpn	CCC-GGTACC(KpnI)-TGAAGAAGTACCTGAAGAAG
0995SOE2-Kas	GAAATCTCCAACTACCATGTT-GGCGCC(KasI)-GTTTTTGCCCTCCTAAGATT
0995SOE3-Kas	AATCTTAGGAGGGCAAAAAC-GGCGCC(KasI)-AACATGGTAGTTGGAGATTTC
0995SOE4-Sac	CCC-GAGCTC(SacI)-TACTTCTTGTAAGTTTAAAGCA
1305SOE1-Kpn	CCC-GGTACC(KpnI)-TGCACAAGCGGCTAGTTTA
1305SOE2-Kas	TAAACTATTTTGTGTTGTGGA-GGCGCC(KasI)-GACTTATTTCCCCCTAGTTA
1305SOE3-Kas	TAACTAGGGGGAAATAAGTC-GGCGCC(KasI)-TCCACAACACAAAATAGTTTA
1305SOE4-Sac	CCC-GAGCTC(SacI)-AATCATAAATTATAGAATATCGG
1464SOE1-Kpn	CCC-GGTACC(KpnI)-GCTAAAGGGCTTTTATTATCA
1464SOE2-Kas	ATAGATGCATCTATGTTATCA-GGCGCC(KasI)-ACTTTCCCTCCCTAGAATT
1464SOE3-Kas	AATTCTAGGGAGGGAAAGT-GGCGCC(KasI)-TGATAACATAGATGCATCTAT
1464SOE4-Sac	CCC-GAGCTC(SacI)-GCTGGTCTTGCGATACCA
KanF-Kas	TCCC-GGCGCC(KasI)-CTCGACGATAAACCCAGCGAAC
KanR-Kas	TCCC-GGCGCC(KasI)-CTTTTTAGACATCTAAATCTAGGTAC
0829CompF-Bam	GGC-GGATCC(BamHI)-GTAAGATTATTGGATTTTCATTT
0829CompR-EcoRI	GGC-GAATTC(EcoRI)-CTATCTATGACAATGAAAGG
UniCompSOE1-Pst	ATAT-CTGCAG(PstI)-ATCCCATTATGCTTTGGCA
0328CompSOE2	AATCGGTTCTATTAAGTACATGGGTTTCACTCTCCTTCTA
0328CompSOE3	TAGAAGGAGAGTGAAACCCATGTACTTAATAGAACCGATT
0328CompSOE4-Sac	ATAT-GAGCTC(SacI)-TAGAAACAAAAACCATATATATA
0930CompSOE2	ATTATTATTACTAATGAATTTCATGGGTTTCACTCTCCTTCTA
0930CompSOE3	TAGAAGGAGAGTGAAACCCATGAAATTCATTAGTAATAATAAT
0930CompSOE4-Sac	ATAT-GAGCTC(SacI)-ACTATTCTTAACATCTCATAC
1494CompSOE2	AAAATTCCAAGTTTCAGTCATGGGTTTCACTCTCCTTCTA
1494CompSOE3	TAGAAGGAGAGTGAAACCCATGACTGAAACTTGGAATTTT
1494CompSOE4-Sac	ATAT-GAGCTC(SacI)-TGTAGGTAAAATAGTCTACG
0571CompSOE2	CGAATGCGAAAGATTGCATGGGTTTCACTCTCCTTCTA
0571CompSOE3	TAGAAGGAGAGTGAAACCCATGCAATCTTTCGCATTCG
0571CompSOE4-Sac	ATAT-GAGCTC(SacI)-AAAAATAACAGCCCCAAACG
ptaSOE1-Pst	ATAT-CTGCAG(PstI)-ATCCCATTATGCTTTGGCA
ptaSOE2	ATACATTTAATAAATCAGCCATGGGTTTCACTCTCCTTCTA
ptaSOE3	TAGAAGGAGAGTGAAACCCATGGCTGATTTATTAAATGTAT
ptsSOE4	ATAT-GAGCTC(SacI)-TTATTGTAAGGCTTGCGCT
pta-0571SOE1-Pst	ATAT-CTGCAG(PstI)-ATCCCATTATGCTTTGGCA
pta-0571SOE2	ATACATTTAATAAATCAGCCATGGGTTTCACTCTCCTTCTA
pta-0571SOE3	TAGAAGGAGAGTGAAACCCATGGCTGATTTATTAAATGTAT
pta0571SOE4-Sac	ATAT-GAGCTC(SacI)-AAAAATAACAGCCCCAAACG

### Generation of in-frame deletion mutants

Briefly, recombinant pIMAY [57] containing 500 bp regions of homology upstream and downstream of the target gene were transformed into *S*. *aureus* LAC via electroporation and propagated at 28–30°C in the presence of chloramphenicol. Plasmid recombination into the chromosome was induced by shifting to 37°C, a non-permissive temperature for pIMAY replication, in the presence of chloramphenicol. Plasmid integrants were then incubated at 30°C without antibiotics, to induce plasmid replication and excision from the chromosome. Bacteria were subsequently plated on medium containing anhydrous tetracycline (AnTet), which induces the expression of a *secY* antisense RNA transcript under control of the Pxyl/tetO promoter and serves as a counter-selection against any *S*. *aureus* still harboring pIMAY [57]. Following counter-selection, chloramphenicol sensitive bacteria were screened for the presence of the desired mutation by PCR. The following mutants were generated using this approach: Δ*lipA*, Δ*lipM*, Δ*lplA1*, Δ*lplA2*, Δ*lipL*, Δ*lipA*Δ*lipM*, Δ*lipA*Δ*lplA1*, Δ*lipA*Δ*lplA2*, Δ*lplA1*Δ*lplA2*, Δ*lipM*Δ*lplA1*, *ΔlipLΔlplA1* and Δ*lipA*Δ*lplA1*Δ*lplA2*.

### Construction of marked deletion mutants

To overcome difficulties encountered when generating mutants with growth deficiencies (*lipL*, *e2-pdh*, *e2-ogdh*, and *gcvH)*, we constructed mutagenesis plasmids containing an antibiotic resistance marker, *aphA3 (kan* ^*R*^ *)*. The *kan* ^R^ resistance gene was PCR amplified from plasmid pBTK [69] with oligonucleotides TCCC-GGCGCC-CTCGACGATAAACCCAGCGAAC and TCCC-GGCGCC-CTTTTTAGACATCTAAATCTAGGTAC and sub-cloned into a unique KasI site engineered between the upstream and downstream regions of homology previously cloned into pIMAY. Once constructed, the pIMAY gene replacement constructs were transformed into wild type *S*. *aureus* LAC as described above. Allelic replacement was carried out as already described and gene replacement mutants were selected on Kan/Neo plates. This procedure was used to generate the following mutant strains: Δ*lipL*::*kan*, Δ*e2-pdh*::*kan*, Δ*e2-ogdh*::*kan*, and Δ*gcvH*::*kan*.

### Construction of *lipL* markerless deletion mutant

Our first attempts to construct an unmarked deletion of *lipL* failed to yield mutants, likely due growth defects associated with a role for *lipL* in supporting essential metabolic pathways in *S*. *aureus*. To generate this growth defective mutant strain, we constructed the above-mentioned gene replacement mutant carrying an antibiotic selection marker (*aphA3*) in place of *lipL*. This initial strain, Δ*lipL*::*kan*, was first transduced into the parental LAC strain to isolate an independent Δ*lipL*::*kan* mutant. The newly transduced strain was then used to construct a Δ*lipL* unmarked deletion mutant. The removal of the antibiotic resistance gene was achieved using pIMAY containing *lipL* upstream and downstream 500 bp regions of homology, constructed as described above leading to the generation of a Δ*lipL* mutant strain.

### Construction of Δ*lipA*Δ*lipL*, Δ*lipM*Δ*lipL*, Δ*lipL*Δ*lplA2*, Δ*lipM*Δ*lplA2*, and Δ*lipL*Δ*lipA*Δ*lipM*

Δ*lipL*::*kan* was introduced into Δ*lipA*, Δ*lipM* and Δ*lplA2* mutant backgrounds via generalized transduction using bacteriophage ϕ11 (See methods below). Δ*lipM*Δ*lplA2* was constructed by transducing a *lplA2*::*erm* mutation into a Δ*lipM* mutant background using bacteriophage ϕ11. *lplA2*::*erm* (NE266) was obtained from the University of Nebraska *S*. *aureus* USA300 *bursa aurealis* transposon mutant library [70]. Δ*lipL*Δ*lipA*Δ*lipM* was generated by transducing *S*. *aureus* LAC Δ*lipA* with a *lipM*::*erm* transposon insertion mutant, obtained from NE1334 (*lipM*::*erm*) [70], as well as the *lipL* gene replacement mutant bearing a kanamycin resistance cassette (Δ*lipL*::*kan*).

### Bacteriophage-mediated generalized transduction

*S*. *aureus* bacteriophage ϕ11 was used for all transductions. To package phage with donor DNA, a 3 mL overnight culture of the donor strain containing the marked gene of interest was incubated with shaking at 37°C in TSB-LB media (mixed 1:1) supplemented with 5 mM CaCl_2_ and 5 mM MgSO_4_ (Amresco). The overnight culture was diluted 1:100 in 10 mL of TSB-LB media supplemented with 5 mM CaCl_2_ and 5 mM MgSO_4_, and was then grown at 37°C in a shaking incubator for 2.5–3 hours, until reaching a OD 600 nm of 0.3 to 0.9. Upon reach the desired optical density, 500 μL of bacterial culture was incubated at room temperature with 10-fold serial dilutions of ϕ11 phage lysate stock in TMG buffer (10 mM Tris pH 7.5, 5 mM MgCl_2_, 0.01% gelatin (v/v)), aiming to achieve an approximate multiplicity of infection of 1:1. After 30 minutes, the tubes containing the bacterial suspension and the phage dilutions were mixed with 3 mL of CY Top agar (casamino acids 5 g/L, yeast extract 5 g/L glucose 5 g/L NaCl 6 g/L, 7.5 g/L agar. +/- BCFA as needed) (Amresco), cooled at 55°C, and supplemented with 5 mM CaCl_2_ and 5 mM MgSO_4_ and immediately poured onto TSA (+/-BCFA as needed) plates. Once solidified, plates were incubated overnight at 30°C. Following overnight growth, the top agar of 2–3 plates was scraped from those with confluent plaques using a sterile scoopula and introduced into a single 50 mL tube. The top agar was then suspended in 2 mL of phage buffer (TMG) per plate worth of top agar. After extensive vortexing, the tubes were centrifuged for 15 minutes at maximum speed and the supernatant was filter-sterilized twice using 0.45 μm and 0.22 μm filters. Phage stocks were stored at 4°C.

To transduce recipient strains, overnight cultures containing 20 mL of the recipient cells in TSB:LB supplemented with 5 mM CaCl_2_ were centrifuged at maximum speed for 15 minutes and resuspended in 3 mL TSB:LB media (1:1), also supplemented with 5mM CaCl_2_. Phage infection was carried out by incubating 500 μL serial dilutions (1; 1:10; 1:100) of the bacterial suspension with either 100 μL of ϕ11 phage (10^8^−10^9^ PFU) or 100 μL of TMG buffer (control uninfected) at room temperature for 30 minutes, inverting the tubes every 10 minutes. After incubation, 40 mM final sodium citrate was added to the tubes from a 1 M stock solution and samples were incubated for an additional 30 minutes, inverting tubes every 10 minutes. Samples were then centrifuged at maximum speed for 5 minutes and washed in 500 μL LB:TSB medium supplemented with 40mM sodium citrate. After a second centrifugation step, cells were resuspended in 200 μL TSB containing 40 mM sodium citrate and 100 μL was spread onto TSA BCFA plates containing 10 mM sodium citrate supplemented with the antibiotic of interest. Plates were incubated at 30°C for 24–48 hours or until detection of bacterial colonies. Potential transductants were later validated via PCR.

### Generation of complementation strains

*S*. *aureus* complementation strains were generated using plasmid pJC1111, except for complementation with *lipA*, which used the vector pJC1112 [68, 71]. Both pJC1111 and pJC1112 stably integrate into the SaPI-1 site of the *S*. *aureus* chromosome after passage through strain *S*. *aureus* RN9011 containing pRN7203 expressing SaPI-1 integrase, leading to single-copy stable integration of the plasmid [68].

In order to construct complementation strains we first generated pJC1111 plasmids that drive expression of the *lipM*; *lipL*; *pta*; *pta-lipL*; *lplA1* or *lplA2* genes under the control of the constitutive *P*_HELP_ promoter obtained from pIMAY [56–58]. All oligonucleotides designed for amplification are listed in Table 2. Primers UniCompSOE1-Pst / ptaSOE1-Pst / pta-0571SOE1-Pst and 1494CompSOE2 / 0571CompSOE2 / 0328CompSOE2 / 0930CompSOE2 / ptaSOE2 / pta-0571SOE2 were used to amplify the *P*_HELP_ promoter using pIMAY as template DNA. Primers 1494CompSOE3 / 0571CompSOE3 / 0328CompSOE3 / 0930CompSOE3 / ptaSOE3 / pta-0571SOE3 along with primers 1494CompSOE4-Sac / 0571CompSOE4-Sac / 0328CompSOE4-Sac / 0930CompSOE4-Sac / ptaSOE4-Sac / pta-0571SOE4-sac were used to amplify the open reading frame corresponding to the gene of interest for complementation including ~150 nucleotides downstream of the stop codon. These two DNA fragments were joined by SOEing PCR as described above. All amplicons were subsequently purified and cloned into pJC1111 after digestion with restriction enzymes Pst1 and Sac1 and subsequent ligation generating pJC1111-*lipM;* pJC1111-*lipL*, pJC1111-*lplA1*, pJC1111-*lplA2*, pJC1111-*pta* and pJC1111-*pta-lipL*.

All complementation plasmids were propagated in *E*. *coli* DH5α, followed by isolation and subsequent electroporation into *S*. *aureus* RN9011 and plating on TSA+BCFA plates supplemented with chloramphenicol and CdCl_2._ Plasmid integrants were used as donors to package and transduce the complementation allele into the desired LAC mutant strain background. Final complementation strains were selected based on their CdCl_2_ resistance and validated via PCR. This resulted in the generation of strains Δ*lipM+lipM;* Δ*lipL+lipL;* Δ*lipL+pta;* Δ*lipL+pta-lipL;* Δ*lipA*Δ*lipM+lipM*, Δ*lipA*Δ*lplA1+lplA1;* Δ*lipA*Δ*lplA2+lplA2*, Δ*lplA1*Δ*lplA2+lplA1*, Δ*lplA1*Δ*lplA2+lplA2*, Δ*lipA*Δ*lplA1*Δ*lplA2+lplA1 and* Δ*lipA*Δ*lplA1*Δ*lplA2+lplA2*.

Complementation of Δ*lipA* was accomplished by amplification of the *lipA* gene with its putative native promoter region using primers 0829CompF-Bam and 0829CompR-EcoRI. The resultant amplicon was cloned into pJC1112 after digestion with enzymes BamHI and EcoRI. pJC1112-*lipA* was subsequently electroporated into *S*. *aureus* RN9011. The integrated complementation vector was later packaged into bacteriophage ϕ11 and transduced into a Δ*lipA* mutant background. Positive transductants were selected based on their erythromycin resistance and designated Δ*lipA+lipA*.

### Evaluation of the requirement of lipoic acid or octanoic acid for growth in vitro

Bacterial growth curves were carried out in six different media: RPMI, RPMI + BCFA, RPMI + lipoic Acid (LA), RPMI + octanoic acid (OA), RPMI + 20% fetal bovine serum (FBS), and RPMI + LA + 20% FBS. Overnight cultures were prepared in triplicate from three individual colonies by inoculating 200 μL of RPMI+BCFA in adjacent wells of a round-bottom 96-well polystyrene plate (Corning) followed by incubating overnight with shaking at 200 rpm at 37°C. The following morning, cells were pelleted at 3,700 rpm for 15 minutes and washed three times with 200 μL of RPMI to remove any remaining branched chain carboxylic acids. After washing cells, 2 μL of each triplicate sample was inoculated into flat-bottom polystyrene 96-well plates (Corning) containing 198 μL of RPMI, RPMI+BCFA, RPMI+25 nM α-lipoic acid (Sigma), RPMI+250 μM octanoic acid (Sigma), RPMI + 20% FBS, and RPMI + 25 nM α-Lipoic acid + 20% FBS. Bacterial growth was assessed over time by measuring optical density at 550 nm (OD550) using an ELx800 microplate reader (BioTek) until reaching stationary phase (9–10 hours). Results were analyzed and graphed using Prism (GraphPad Software, Inc., San Diego, CA). All growth curves were conducted at least three times in triplicate. The mean optical density at 550 nm and standard deviation are shown for all curves. In all cases where error bars are not seen, the standard deviation was sufficiently small such that figure symbols prevented their display.

### Whole cell lysate preparation

*S*. *aureus* wild type and mutant strains were grown overnight with shaking at 37°C in 15 mL conical tubes containing 5 mL of RPMI+BCFA media. A subculture of 60 μL of these samples was inoculated into 15 mL conical tubes containing 6 mL of RPMI+BCFA; RPMI+BCFA+5 μM α-lipoic acid, RPMI + BCFA+170 μM octanoic acid, RPMI + BCFA + 5 μM α-lipoic acid, or RPMI+BCFA + 5 μM α-lipoic acid + 20% FBS. Samples were incubated with shaking at 200 rpm for 9 hours and bacterial growth was determined by measuring optical density at 600 nM using a Genesys 10S UV-Vis spectrophotometer (Thermo). The remaining culture volume was centrifuged at 4,200 rpm for 15 minutes, the supernatant was discarded, and the bacterial pellets were stored at -80°C until whole cell lysates were prepared. After thawing frozen pellets on ice, the bacteria were suspended in 250 μL of PBS and transferred to screw cap microcentrifuge lysing tubes (Fisher Scientific) containing 250 μL of 0.1 mm glass cell disruption beads (Scientific Industries, Inc.). Cells were lysed using a Fast Prep-24 5G (MP Biomedicals) bead disruption system in two sequential steps, at 5.0 speed for 20 seconds and at 4.5 speed for 20 seconds, each separated by a 5 minute incubation period on ice. After cell disruption, samples were centrifuged at 13,000 rpm for 15 minutes. 130 μL of the supernatant were collected in microcentrifuge tubes containing 43 μL of 6X SDS sample buffer and subsequently boiled for 10 minutes prior to storage at -20°C.

### Determination of protein lipoylation

Protein samples from OD-normalized whole cell lysates were separated by sodium dodecyl sulfate polyacrylamide gel electrophoresis (SDS-PAGE) in 12% polyacrylamide gels at 120 volts for approximately 4 hours. Coomassie staining was performed to evaluate protein patterns and equivalent loading of samples using GelCode Blue stain reagent (Thermo) with Precision Plus Protein Ladder (Thermo) used as a molecular weight marker. Protein lipoylation was assessed via immunoblot. Briefly, resolved proteins were transferred from polyacrylamide gels to 0.2 μm PVDF membranes (Immobilon, Roche) at 200 V for 1 hour in a Quadra Mini-Vertical PAGE/Blotting System (CBS Scientific). After transfer, membranes were incubated overnight with PBST (0.1% Tween-20 in PBS) supplemented with 5% BSA at 4°C. Nonspecific binding of antibody to *S*. *aureus* antibody binding proteins, Protein A and Sbi, was blocked by incubating the membranes with 0.9 mg/mL human IgG (Sigma) for 1 hour. A 1:3,000 dilution of rabbit polyclonal anti-lipoic acid antibody (Calbiochem) in PBST was added to the membrane followed by incubation for 1 hour and three subsequent washes in ~20 mL of PBST. Goat anti-Rabbit IgG (H+L) HRP conjugate (Thermo) was then added at a 1:200 dilution in PBST for an additional hour followed by 3, 15 minute washes in ~20 mL of PBST. Western blot images were captured on FluorChemE System (Protein Simple) using SuperSignal West Pico Chemiluminiscent Substrate (Thermo).

### Isolation of murine bone marrow derived macrophages and infection with *S*. *aureus*

Murine macrophages were derived from bone marrow progenitor cells isolated from the femurs and tibias of C57Bl/6 mice as previously described [72, 73]. Macrophages were cultured at 37°C, 5% CO2 in DMEM (CellGro) + 1 mM Sodium Pyruvate (CellGro) + 2 mM L-glutamine (CellGro) + 20% FBS (Seradigm). The day before infection with *S*. *aureus*, macrophages were seeded (65,000 cells per well) into 96-well flat-bottom tissue culture treated plates (Corning) in the absence of antibiotic. Overnight cultures of *S*. *aureus* were normalized to the same optical density at 600 nm (OD_600_ ~0.32, ~1.0x10^8^ CFU/mL) and subsequently added to macrophages at a multiplicity of infection of one bacterium to one macrophage. Upon addition of *S*. *aureus*, the 96 well plates were spun for 7 minutes at 1500 RPM to synchronize infection across all wells and immediately placed in a 37°C, 5% CO2 incubator for 30 minutes. After 30 minutes, cells were washed extensively (3X) with 150 μL PBS followed by the addition of medium containing 50 μg/mL gentamicin for an additional 30 minutes. Infected cells were then washed an additional 3X in PBS and placed in medium without antibiotic for the remainder of the infection time course. At T1, T2, T4, T6, and T8 hours, saponin (0.1%) was added to one 96 well plate containing triplicate wells infected with each *S*. *aureus* strain and the plate was placed on ice for 20 minutes. 10-fold serial dilutions of the cell lysate were made and spot plated onto BCFA-containing tryptic soy agar plates to enumerate bacterial CFU.

### Murine systemic infections

All murine infection experiments were performed at least three times with cohorts of at least 4 animals. Overnight cultures of each strain were inoculated from freshly isolated single colonies struck out from the freezer the day prior and grown with shaking at 37°C for ~16 hours. A 1:100 subculture into 15 mL of TSB+BCFA was performed followed by incubation with shaking at 200 rpm, 37°C for 3–4 hours (until reaching optical density at 600 nm near 1.0). Cultures were then centrifuged for 5 minutes at maximum speed in a tabletop centrifuge. Cell pellets were washed twice in 5 mL PBS and 2 mL of the final bacterial suspension was added to a 15 mL conical tube containing 8 mL of PBS. Bacterial suspensions were then normalized with PBS to an OD600 of 0.32–0.33 (1 x 10^8^ CFU/mL). Six to eight week old female Swiss Webster mice obtained from Envigo (formerly Harlan) were used in all experiments. Mice were deeply anesthetized with 2,2,2-tribromoethanol (Avertin) (250 mg/kg) (Sigma), via intraperitoneal injection followed by inoculation with 100 μL PBS containing 1 x 10^7^ CFU wild type *S*. *aureus* or mutant strains directly into the bloodstream via injection into the retro-orbital venous plexus. After infection, the remaining bacterial suspension was plated on TSA-BCFA plates to ensure viability of cells in PBS and accurate infection inoculums. All strains were fully viable in PBS and all animals received between (1.0 and 2.0 x 10^7^ CFU). Infected mice were monitored daily and their kidneys and hearts were isolated at 96 hours post-infection immediately after euthanasia. Tissues were aseptically isolated, homogenized, and spread onto TSA-BCFA medium plates and incubated overnight at 37°C in order to enumerate CFU.

### Statistical analyses

Statistical analyses were performed using Prism GraphPad Software, version 7. For all relevant datasets in this study, a nonparametric 1-way ANOVA with Kruskal-Wallis multiple comparisons post-test was used. *N* values and additional statistical information is provided in the relevant figure legend. All growth curves were conducted a minimum of three times, generated from three independent single colonies, and OD550 values averaged for each experiment. Mean and standard deviations were plotted at each time point. All immunoblots were conducted at least 4 times from freshly prepared OD-normalized whole cell lysates.

### Ethics statement

All animal experiments were performed in ABSL2 facilities with protocols that are approved by Loyola University of Chicago, Health Sciences division Institutional Animal Care and Use Committee (IACUC# 2014049) in accordance with guidelines set forth by the USDA and PHS Policy on Humane Care and Use of Laboratory Animals under the guidance of the Office of Laboratory Animal Welfare (OLAW). Loyola University Chicago, Health Sciences Division has an Animal Assurance on file with the Public Health Service (#A3117-01 approved through 02/28/2018), is a fully AAALAC International accredited institution (#000180, certification dated 11/19/2013), and is a USDA registered/licensed institution (#33-R-0024 through 08/24/2017). Loyola University Chicago, Health Sciences Division’s Institutional Animal Care and Use Committee (IACUC) is responsible for reviewing all protocols involving living vertebrate animals ensuring compliance with federal regulations, inspecting animal facilities and laboratories and overseeing training and educational programs. Mice were anesthetized with 2,2,2-tribromoethanol (Avertin) (250 mg/kg) prior to infection and euthanasia was carried out by C02 narcosis.

## Supporting Information

S1 Fig Amino acid sequence alignment of lipoic acid biosynthesis and salvage enzymes. *Bs–B*. *subtilis*; *Lm–L*. *monocytogenes*; *Sa–S*. *aureus*. All identical amino acids are shown; +, similar amino acids.
(PDF)

S2 Fig A Δ*lipL* mutant requires introduction of the entire *pta-lipL* operon for full complementation. (A) Growth curves of the indicated strains in RPMI. (B) Whole cell lysates of the indicated *S*. *aureus* strains collected after 9 hours of growth in RPMI + BCFA (2-methyl butyric acid, isovaleric acid, isobutyric acid, and sodium acetate) followed by immunoblotting for lipoic acid-containing proteins. (C) Representative coomassie-stained gel of OD normalized cell lysates of the indicated strains. In all growth curves, the mean +/- standard deviation of triplicate data points is shown. In any case where an error bar is not visible, the standard deviation was smaller than the size of the symbol used at that data point.
(TIF)

S3 Fig All lipoic acid biosynthesis and salvage gene deletion mutants replicate in BCFA medium and achieve equivalent final OD in stationary phase. (A-E) Growth curves of the indicated strains in RPMI + BCFA (2-methyl butyric acid, isovaleric acid, isobutyric acid, and sodium acetate). In all growth curves, the mean +/- standard deviation of triplicate data points is shown. In any case where an error bar is not visible, the standard deviation was smaller than the size of the symbol used at that data point.
(TIF)

S4 Fig Coomassie-stained gels of OD-normalized cell lysate proteins from BCFA-grown cultures used in immunoblots. Representative coomassie-stained gels of OD normalized cell lysates of the indicated strains. Samples correspond to those used in immunoblots in Figs 2, 4 and 6.
(TIF)

S5 Fig Identification of lipoylated E2 and H subunits in *S*. *aureus*. Whole cell lysates of the indicated *S*. *aureus* strains collected after 9 hours of growth in RPMI + BCFA (2-methyl butyric acid, isovaleric acid, isobutyric acid, and sodium acetate) + lipoic acid (LA), followed by immunoblotting for lipoic acid-containing proteins.
(TIF)

S6 Fig A Δ*lipM* mutant is less efficient at generating lipoyl proteins when free octanoic acid is present. Whole cell lysates of the indicated *S*. *aureus* strains collected after 9 hours of growth in RPMI + BCFA (2-methyl butyric acid, isovaleric acid, isobutyric acid, and sodium acetate) + octanoic acid (OA), followed by loading 1.5X the amount of sample and immunoblotting for lipoic acid-containing proteins.
(TIF)

S7 Fig Coomassie-stained gels of OD-normalized cell lysate proteins from BCFA-grown cultures used in immunoblots. Representative coomassie-stained gel of OD normalized cell lysates of the indicated strains. Samples correspond to those used in immunoblots in Figs 7 and 8.
(TIF)
