# Supplementary material for: Staphylococcus aureus Tissue Infection During Sepsis Is Supported by Differential Use of Bacterial or Host-Derived Lipoic Acid
Source: PLoS Pathog. 2016 Oct 4;12(10):e1005933. doi: 10.1371/journal.ppat.1005933 (PMC5049849; doi:10.1371/journal.ppat.1005933)
Supplement: S1 Fig — Bs–B. subtilis; Lm–L. monocytogenes; Sa–S. aureus. All identical amino acids are shown; +, similar amino acids. (PDF) [file ppat.1005933.s001.pdf]

Figure S1. Amino Acid sequence alignment of lipoic acid biosynthesis and salvage enzymes.
Bs – B. subtilis; Lm – L. monocytogenes; Sa – S. aureus

```
Bs LipA  1    MAKKDEH-LRKPEWLKIKLNTNENYTGLKKLMRENNLHTVCEEAKCPNIHECWAVRRTAT  59
              MA K+E  LRKP+WLKIKLNTNENYTGLKK+MRE NL+TVCEEAKCPNIHECW  RRTAT
Sa LipA  1    MATKNEEILRKPDWLKIKLNTNENYTGLKKMMREKNLNTVCEEAKCPNIHECWGARRTAT  60

Bs LipA  60   FMILGSVCTRACRFCAVKTGLPTELDLQEPERVADSVALMNLKHAVITAVARDDQKDGGA  119
              FMILG+VCTRACRFCAVKTGLP ELDL EPERVA+SV LMNLKH VITAVARDD +D G+
Sa LipA  61   FMILGAVCTRACRFCAVKTGLPNELDLNEPERVAESVELMNLKHVVITAVARDDLRDAGS  120

Bs LipA  120  GIFAETVRAIRRKSPFTTIEVLPSDMGGNYDNLKTLMDTRPDILNHNIETVRRLTPRVRA  179
               ++AETVR +R ++PFTTIE+LPSDMGG+YD L+TLM +RPDILNHNIETVRRLTPRVRA
Sa LipA  121  NVYAETVRKVRERNPFTTIEILPSDMGGDYDALETLMASRPDILNHNIETVRRLTPRVRA  180

Bs LipA  180  RATYDRSLEFLRRAKEMQPDIPTKSSIMIGLGETKEEIIEVMDDLLANNVDIMAIGQYLQ  239
              RATYDR+LEFLRR+KE+QPDIPTKSSIM+GLGET EEI E MDDL AN+VDI+ IGQYLQ
Sa LipA  181  RATYDRTLEFLRRSKELQPDIPTKSSIMVGLGETIEEIYETMDDLRANDVDILTIGQYLQ  240

Bs LipA  240  PTKKHLKVQKYYHPDEFAELKEIAMQKGFSHCEAGPLVRSSYHADEQVNEASKKRQAQA   298
              P++KHLKVQKYY P EF +L+++AM KGF HC+AGPLVRSSYHADEQVNEA+K++Q Q
Sa LipA  241  PSRKHLKVQKYYTPLEFGKLRKVAMDKGFKHCQAGPLVRSSYHADEQVNEAAKEKQRQG   299
```

79% Identity

```
Bs LipM  4    ETWRFIDSGNASPAFNMALDEALLYWHSEKKIPPVIRFYGWNPATLSVGYFQNIKKEINF  63
              ETW FI++G+  P +NMA+DEALL + S  +I PVIRFY WNPATLS+GYFQ ++KEI+
Sa LipM  3    ETWNFINTGSKDPYYNMAMDEALLNFVSRGEIDPVIRFYTWNPATLSIGYFQRLQKEIDI  62

Bs LipM  64   EAVHKYNLGFVRRPTGGRGVLHDQELTYSVIVSEEHPEMPATVTEAYRVISEGILQGFRN  123
              + V +   G VRR TGGRGVLHD+ELTYSVIV E HP MP+TVTEAYRVIS+G+L+GF+N
Sa LipM  63   DKVKEKGFGLVRRQTGGRGVLHDKELTYSVIVPESHPNMPSTVTEAYRVISQGLLEGFKN  122

Bs LipM  124  LGLDAYFAIPRTEKEKESLKNPRSSVCFDAPSWYELVVEGRKVAGSAQTRQKGVILQHGS  183
              LG D YFA+P+T +E++ LK PRSSVCFDAPSWYELVVEGRK+AGSAQTRQKGVILQHGS
Sa LipM  123  LGFDTYFAVPKTPEERQKLKQPRSSVCFDAPSWYELVVEGRKIAGSAQTRQKGVILQHGS  182

Bs LipM  184  ILLDLDEDKLFDLFLYPSERVRERMQRNFKNKAVAINELIEKRVTMDEARKAFKEGFETG  243
              IL D+D D+LFD+F+Y +ER++ +M+  F  KAVAIN++ ++ +T+ +  +AF++GF+ G
Sa LipM  183  ILQDIDIDELFDMFIYKNERLKLKMKEAFVEKAVAINDISDEHITISQMEEAFEKGFKKG  242

Bs LipM  244  LNIHLEPYELSQEELDFVHHLAETKYASDEWNYKR  278
              LNI  +P EL++ +L  V  L E KY SDEW +++          62% Identity
Sa LipM  243  LNIEFKPLELTEAQLAEVEELTE-KYRSDEWMFRK  276
```

```
Bs LipL  30   QSFAMDDTLCMSVGKGVSPATARSWVHHDTIVLGIQDTRLPFLQDGISLLESE-GYRVIV  88
              QSFA DDT C SVGK +S    R+W+H  T++LGI D+RLPFL+DGI  L +E GY  IV
Sa LipL  2    QSFAFDDTFCESVGKDISDNVVRTWIHQHTVILGIHDSRLPFLKDGIDYLTNEIGYNAIV  61

Bs LipL  89   RNSGGLAVVLDDGVLNISLIFEDEKKGIDIDKGYEAMVELMRRMLRPYNAKIEAYEIEGS  148
              RNSGGL VVLD GVLNISL+F+ + +   ID+ +   M  L+ +M    N  I+  EIE S
Sa LipL  62   RNSGGLGVVLDQGVLNISLMFKGQTE-TTIDEAFTVMYLLISKMFENENVDIDTMEIEHS  120

Bs LipL  149  YCPGSYDLSINGKKFAGISQRRVRGGVAVQIYLCADKSGSERADLIRRFYQAALKDKQND  208
              YCPG +DLSI+GKKFAGISQRRVRGG+AVQIYLC + SGSERA +++ FY+ ALK +
Sa LipL  121  YCPGKFDLSIDGKKFAGISQRRVRGGIAVQIYLCVEGSGSERALMMQTFYEHALKGEVTK  180

Bs LipL  209  KKGVYPEIRPETMASLSELLQKDISVQDLMFALLTELKALSTHLYSAGLSIDEEMEFEKN  268
               K  YPEI P  MASL  LL K I+VQD+MF LL  +K L   L    ++ +E   ++
Sa LipL  181  FK--YPEIEPSCMASLETLLNKTITVQDVMFLLLYAIKDLGGVLNMTPITQEEWQRYDTY  238

Bs LipL  269  LVRMAERNAKV  279
                +M ERN K+        54% Identity
Sa LipL  239  FDKMIERNKKM  249
```

Figure S1. Amino Acid sequence alignment of lipoic acid biosynthesis and salvage enzymes.
Bs – B. subtilis; Lm – L. monocytogenes; Sa – S. aureus

```
Bs LplJ   1    MLFIDNQNINDPRINLAIEEYCVKHLDPEQQYLLFYVNQPSIIIGKNQNTIEEINTKYVE  60
               M FI N NI DP +NLA+EEY +K+L  E+ Y LFY+N+PSII+GKNQNTIEE+N  Y++
Sa LplA1 1    MKFISNNNITDPTLNLAMEEYVLKNLPAEESYFLFYINRPSIIVGKNQNTIEEVNQTYID  60

Bs LplJ   61   ENGIIVVRRLSGGGAVYHDLGNLNFSFITKDDGDSFHNFKKFTEPVIQALHQLGVEAELS  120
                + I VVRR+SGGGAVYHD GNLNFSFIT DDG+SFHNF+KFTEP++QAL  LGV AEL+
Sa LplA1 61   AHNIDVVRRISGGGAVYHDTGNLNFSFITDDDGNSFHNFQKFTEPIVQALQSLGVNAELT  120

Bs LplJ   121  GRNDIVVDGRKISGNAQFATKGRIFSHGTLMFDSAIDHVVSALKVKKDKIESKGIKSIRS  180
               GRNDI V   KISGNA    K R+FSHGTLM +S +D V +ALKV   KI+SKGIKS+R
Sa LplA1 121  GRNDIQVGQAKISGNAMVKVKNRMFSHGTLMLNSDLDEVQNALKVNPAKIKSKGIKSVRK  180

Bs LplJ   181  RVANISEFLDDKMTTEEFRSHLLRHIFNTNDVGNVPEYKLTEKDWETIHQISKERYQNWD  240
               RVANI EFL+D +   EEF+  +L+ IF   +   V EYKLT++DWE I ++S ++Y+ W+
Sa LplA1 181  RVANIQEFLNDPLEIEEFKKIILKTIFGETE---VEEYKLTDEDWENIEKLSNDKYRTWE  237

Bs LplJ   241  WNYGRSPKFNLNHSKRYPVGSIDLHLEVKKGKIEDCKIFGDFFGVGDVSEIENLLVGKQY  300
               WNYGR+PK+N    +++  G + +  +VK+GKIE  KIFGDFFGVGDV+++EN LVG  +
Sa LplA1 238  WNYGRNPKYNFEREEKFEKGFVQIKFDVKRGKIEHAKIFGDFFGVGDVTDLENALVGCLH  297

Bs LplJ   301  ERSVIADVLEGVNLKHYFGNITKEDFLDLI  330
               +   I + L   +L HYFG+I + + + L+           57% Identity
Sa LplA1 298  DFEHIEEALSEYDLYHYFGDIDRHELIRLM  327


Bs LplJ   9    INDPRINLAIEEYCVKHLDPEQQYLLFYVNQPSIIIGKNQNTIEEINTKYVEENGIIVVR  68
               I D  I LA++ Y  +H+  ++  L  Y   P + IG+ QNT  E+N  Y++++ I VVR
Sa LplA2 13   ITDGAIALAMQVYVNQHIFLDEDILFPYYCDPKVEIGRFQNTAIEVNQDYIDKHSIQVVR  72

Bs LplJ   69   RLSGGGAVYHDLGNLNFSFITKDDGDSFHNFKKFTEPVIQALHQLGV-EAELSGRNDIVV  127
               R +GGGAVY D G +N   I + D   + +F++F +P I+ALH LG  +   SGRND+ +
Sa LplA2 73   RDTGGGAVYVDKGAVNMCCILEQDTSIYGDFQRFYQPAIKALHTLGATDVVQSGRNDLTL  132

Bs LplJ   128  DGRKISGNAQFATKGRIFSHGTLMFDSAIDHVVSALKVKKDKIESKGIKSIRSRVANISE  187
               +G+K+SG A       RI+   +L+ D   + +   LK  + KI SKGIKS+R+RV ++ E
Sa LplA2 133  NGKKVSGAAMTLMNNRIYGGYSLLLDVNYEAMDKVLKPNRKKIASKGIKSVRARVGHLRE  192

Bs LplJ   188  FLDDK---MTTEEFRSHLLRHIFNTNDVGNVPEYKLTEKDWETIHQISKERYQNWDWNYG  244
                LD+K   +T EEF++ ++  I   +D+     Y+LT+ DWE I +++ ++Y+NWDWNYG
Sa LplA2 193  ALDEKYRDITIEEFKNLMVTQILGIDDIKEAKRYELTDADWEAIDELADKKYKNWDWNYG  252

Bs LplJ   245  RSPKFNLNHSKRYPVGSIDLHLEVKKGKIEDCKIFGDFFGVGDVSEIENLLVGKQYERSV  304
               +SPK+  N S+R   G++D+ + V++ +I DC+I+GDFFG GD+ ++E  L G +  R
Sa LplA2 253  KSPKYEYNRSERLSSGTVDITISVEQNRIADCRIYGDFFGQGDIKDVEEALQGTKMTRED  312

Bs LplJ   305  IADVLEGVNLKHYFGNITKEDFLDLI  330
               +   L+ +++ +YF N+T E  +D+I                   39% Identity
Sa LplA2 313  LTHQLKQLDIVYYFSNVTVEALVDMI  338
```

Figure S1. Amino Acid sequence alignment of lipoic acid biosynthesis and salvage enzymes.
Bs – B. subtilis; Lm – L. monocytogenes; Sa – S. aureus

```
Lm LplA1  1    MYFIDNNNEKDPRINLAVEEFILTELNLDEPVLLFYINKPSIIIGRNQNTVEEIDTEYVE  60
               M FI NNN  DP +NLA+EE++L  L  +E   LFYIN+PSII+G+NQNT+EE++  Y++
Sa LplA1  1    MKFISNNNITDPTLNLAMEEYVLKNLPAEESYFLFYINRPSIIVGKNQNTIEEVNQTYID  60

Lm LplA1  61   KNDVIVVRRLSGGGAVYHDEGNLNFSFITEDDGESFHNFAKFTQPIVEALKRLGVNAELK  120
                +++ VVRR+SGGGAVYHD GNLNFSFIT+DDG SFHNF KFT+PIV+AL+ LGVNAEL
Sa LplA1  61   AHNIDVVRRISGGGAVYHDTGNLNFSFITDDDGNSFHNFQKFTEPIVQALQSLGVNAELT  120

Lm LplA1  121  GRNDLLIDGFKVSGNAQFATKGKMFSHGTLMYDLNLDNVAASLKPRKDKIESKGIKSVRS  180
               GRND+ +   K+SGNA    K +MFSHGTLM + +LD V  +LK    KI+SKGIKSVR
Sa LplA1  121  GRNDIQVGQAKISGNAMVKVKNRMFSHGTLMLNSDLDEVQNALKVNPAKIKSKGIKSVRK  180

Lm LplA1  181  RVANISDFMDQEMTTEEFRDLLLLYIFGVEKVEDVKEYKLTAADWEKIHEISAKRYGNWD  240
               RVANI +F++  +  EEF+ ++L  IFG  +VE   EYKLT  DWE I ++S  +Y  W+
Sa LplA1  181  RVANIQEFLNDPLEIEEFKKIILKTIFGETEVE---EYKLTDEDWENIEKLSNDKYRTWE  237

Lm LplA1  241  WNYGKSPKFDLTRTKRFPVGAVDVRLNVQKGVITDIKIFGDFFGVKNVADIEEKLVNTTY  300
               WNYG++PK++  R ++F  G V ++ +V++G I   KIFGDFFGV +V D+E  LV   +
Sa LplA1  238  WNYGRNPKYNFEREEKFEKGFVQIKFDVKRGKIEHAKIFGDFFGVGDVTDLENALVGCLH  297

Lm LplA1  301  KREVLAEALVDIDVKEYFGNITKDEFLDLL  330
                 E + EAL + D+  YFG+I + E + L+             53% Identity
Sa LplA1  298  DFEHIEEALSEYDLYHYFGDIDRHELIRLM  327
```

```
Lm LplA1  1    MYFID--NNNE--KDPRINLAVEEFILTELNLDEPVLLFYINKPSIIIGRNQNTVEEIDT  56
               MY I+   N E   D  I LA++ ++   + LDE +L  Y   P + IGR QNT  E++
Sa LplA2  1    MYLIEPIRNGEYITDGAIALAMQVYVNQHIFLDEDILFPYYCDPKVEIGRFQNTAIEVNQ  60

Lm LplA1  57   EYVEKNDVIVVRRLSGGGAVYHDEGNLNFSFITEDDGESFHNFAKFTQPIVEALKRLGVN  116
               +Y++K+ + VVRR +GGGAVY D+G +N   I E D   + +F +F QP ++AL  LG
Sa LplA2  61   DYIDKHSIQVVRRDTGGGAVYVDKGAVNMCCILEQDTSIYGDFQRFYQPAIKALHTLGAT  120

Lm LplA1  117  AELK-GRNDLLIDGFKVSGNAQFATKGKMFSHGTLMYDLNLDNVAASLKPRKDKIESKGI  175
                 ++ GRNDL ++G KVSG A      +++   +L+ D+N + +   LKP + KI SKGI
Sa LplA2  121  DVVQSGRNDLTLNGKKVSGAAMTLMNNRIYGGYSLLLDVNYEAMDKVLKPNRKKIASKGI  180

Lm LplA1  176  KSVRSRVANISDFMDQ---EMTTEEFRDLLLLYIFGVEKVEDVKEYKLTAADWEKIHEIS  232
               KSVR+RV ++ + +D+   ++T EEF++L++  I G++ +++ K Y+LT ADWE I E++
Sa LplA2  181  KSVRARVGHLREALDEKYRDITIEEFKNLMVTQILGIDDIKEAKRYELTDADWEAIDELA  240

Lm LplA1  233  AKRYGNWDWNYGKSPKFDLTRTKRFPVGAVDVRLNVQKGVITDIKIFGDFFGVKNVADIE  292
                K+Y NWDWNYGKSPK++  R++R   G VD+ ++V++  I D +I+GDFFG  ++ D+E
Sa LplA2  241  DKKYKNWDWNYGKSPKYEYNRSERLSSGTVDITISVEQNRIADCRIYGDFFGQGDIKDVE  300

Lm LplA1  293  EKLVNTTYKREVLAEALVDIDVKEYFGNITKDEFLDLL  330
               E L  T   RE L   L  +D+  YF N+T +  +D++          41% Identity
Sa LplA2  301  EALQGTKMTREDLTHQLKQLDIVYYFSNVTVEALVDMI  338
```

Figure S1. Amino Acid sequence alignment of lipoic acid biosynthesis and salvage enzymes.
Bs – B. subtilis; Lm – L. monocytogenes; Sa – S. aureus

```
Lm LplA2   1    MIYLDNEDVLDQAYNFAMEEYALRSLDENETYFMFYRMKPTIIVGKNQNTLEEINHPFVK  60
                M ++ N ++ D   N AMEEY L++L   E+YF+FY  +P+IIVGKNQNT+EE+N  ++
Sa LplA1   1    MKFISNNNITDPTLNLAMEEYVLKNLPAEESYFLFYINRPSIIVGKNQNTIEEVNQTYID  60

Lm LplA2   61   DHHIDVLRRLSGGGAVYNDEGNISFSMITKDDGNSFQNFAKFTEPVIRALRKLGVNAELS  120
                 H+IDV+RR+SGGGAVY+D GN++FS IT DDGNSF NF KFTEP+++AL+ LGVNAEL+
Sa LplA1   61   AHNIDVVRRISGGGAVYHDTGNLNFSFITDDDGNSFHNFQKFTEPIVQALQSLGVNAELT  120

Lm LplA2   121  GRNDIEVNGKKISGNAQFATKGRLYSHGTLLFDVDLSMLEKALQVDPEKYLSKGVKSVRS  180
                GRNDI+V   KISGNA    K R++SHGTL+ + DL  ++ AL+V+P K  SKG+KSVR
Sa LplA1   121  GRNDIQVGQAKISGNAMVKVKNRMFSHGTLMLNSDLDEVQNALKVNPAKIKSKGIKSVRK  180

Lm LplA2   181  RVTTIREHLAEDIDILTFKQILLESIFETKDIPRYTFTEADKQGIEKLRTERYRNWDWTY  240
                RV  I+E L + ++I  FK+I+L++IF   ++  Y  T+ D + IEKL  ++YR W+W Y
Sa LplA1   181  RVANIQEFLNDPLEIEEFKKIILKTIFGETEVEEYKLTDEDWENIEKLSNDKYRTWEWNY  240

Lp LplA2   241  GKSPKATIKRKKRFPAGTIEFQVSLEKGQVKEATIYGDFFGTEDVAELAEKIIGCRFERK  300
                G++PK   +R+++F  G ++ +  +++G+++ A I+GDFFG  DV +L   ++GC  + +
Sa LplA1   241  GRNPKYNFEREEKFEKGFVQIKFDVKRGKIEHAKIFGDFFGVGDVTDLENALVGCLHDFE  300

Lm LplA2   301  SIQNAWQEINAKDYFGGIEKEAILDML  327
                 I+ A  E +   YFG I++  ++ ++          47% Identity
Sa LplA1   301  HIEEALSEYDLYHYFGDIDRHELIRLM  327
```

```
Lm LplA2   5    DNEDVLDQAYNFAMEEYALRSLDENETYFMFYRMKPTIIVGKNQNTLEEINHPFVKDHHI  64
                + E + D A   AM+ Y  + +  +E     Y   P + +G+ QNT  E+N  ++  H I
Sa LplA2   9    NGEYITDGAIALAMQVYVNQHIFLDEDILFPYYCDPKVEIGRFQNTAIEVNQDYIDKHSI  68

Lm LplA2   65   DVLRRLSGGGAVYNDEGNISFSMITKDDGNSFQNFAKFTEPVIRALRKLGVNAEL-SGRN  123
                 V+RR +GGGAVY D+G ++   I + D + + +F +F +P I+AL  LG    + SGRN
Sa LplA2   69   QVVRRDTGGGAVYVDKGAVNMCCILEQDTSIYGDFQRFYQPAIKALHTLGATDVVQSGRN  128

Lm LplA2   124  DIEVNGKKISGNAQFATKGRLYSHGTLLFDVDLSMLEKALQVDPEKYLSKGVKSVRSRVT  183
                D+ +NGKK+SG A      R+Y   +LL DV+   ++K L+ + +K  SKG+KSVR+RV
Sa LplA2   129  DLTLNGKKVSGAAMTLMNNRIYGGYSLLLDVNYEAMDKVLKPNRKKIASKGIKSVRARVG  188

Lm LplA2   184  TIREHLAE---DIDILTFKQILLESIFETKDIP---RYTFTEADKQGIEKLRTERYRNWD  237
                 +RE L E   DI I  FK +++  I    DI    RY  T+AD + I++L  ++Y+NWD
Sa LplA2   189  HLREALDEKYRDITIEEFKNLMVTQILGIDDIKEAKRYELTDADWEAIDELADKKYKNWD  248

Lm LplA2   238  WTYGKSPKATIKRKKRFPAGTIEFQVSLEKGQVKEATIYGDFFGTEDVAELAEKIIGCRF  297
                W YGKSPK    R +R  +GT++  +S+E+ ++ +  IYGDFFG  D+ ++ E + G +
Sa LplA2   249  WNYGKSPKYEYNRSERLSSGTVDITISVEQNRIADCRIYGDFFGQGDIKDVEEALQGTKM  308

Lm LplA2   298  ERKSIQNAWQEINAKDYFGGIEKEAILDMLF  328
                 R+ + +  ++++   YF  +  EA++DM+          37% Identity
Sa LplA2   309  TREDLTHQLKQLDIVYYFSNVTVEALVDMIL  339
```
