# Supplementary figures and images for: Staphylococcus aureus Tissue Infection During Sepsis Is Supported by Differential Use of Bacterial or Host-Derived Lipoic Acid
Source: PLoS Pathog. 2016 Oct 4;12(10):e1005933. doi: 10.1371/journal.ppat.1005933 (PMC5049849; doi:10.1371/journal.ppat.1005933)

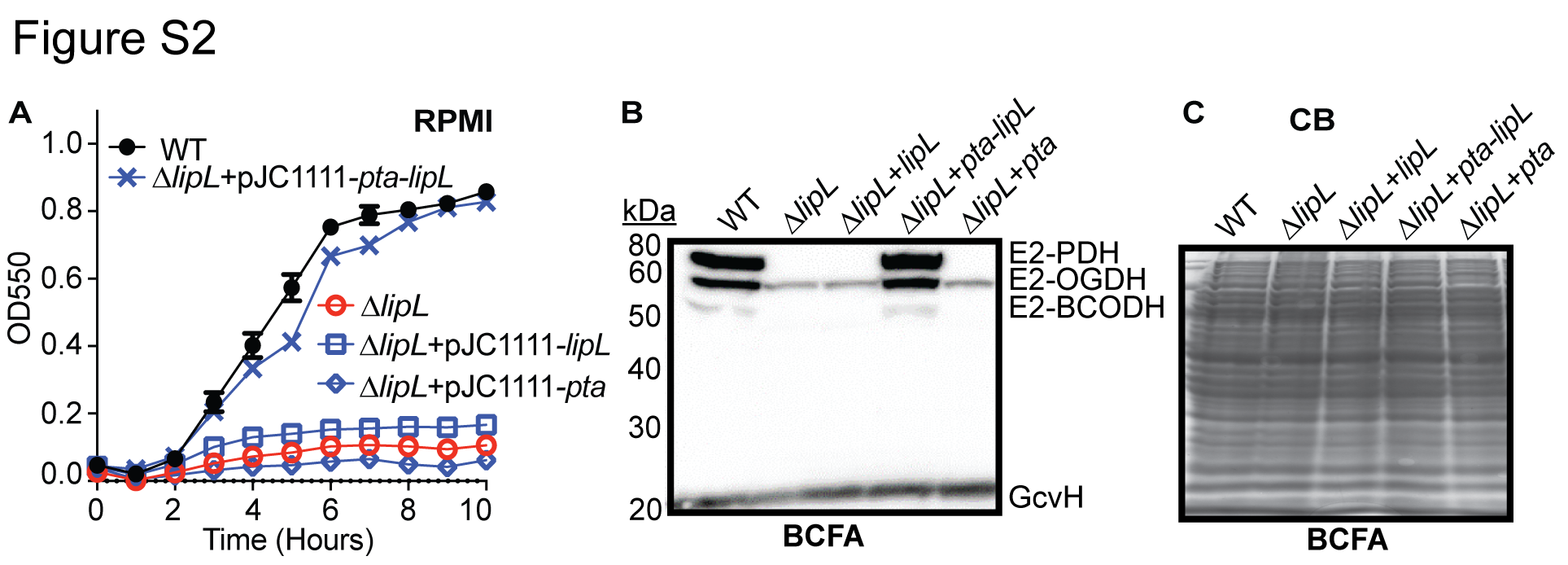

Supplement: S2 Fig — (A) Growth curves of the indicated strains in RPMI. (B) Whole cell lysates of the indicated S. aureus strains collected after 9 hours of growth in RPMI + BCFA (2-methyl butyric acid, isovaleric acid, isobutyric acid, and sodium acetate) followed by immunoblotting for lipoic acid-containing proteins. (C) Representative coomassie-stained gel of OD normalized cell lysates of the indicated strains. In all growth curves, the mean +/- standard deviation of triplicate data points is shown. In any case where an error bar is not visible, the standard deviation was smaller than the size of the symbol used at that data point. (TIF) [file ppat.1005933.s002.tif]

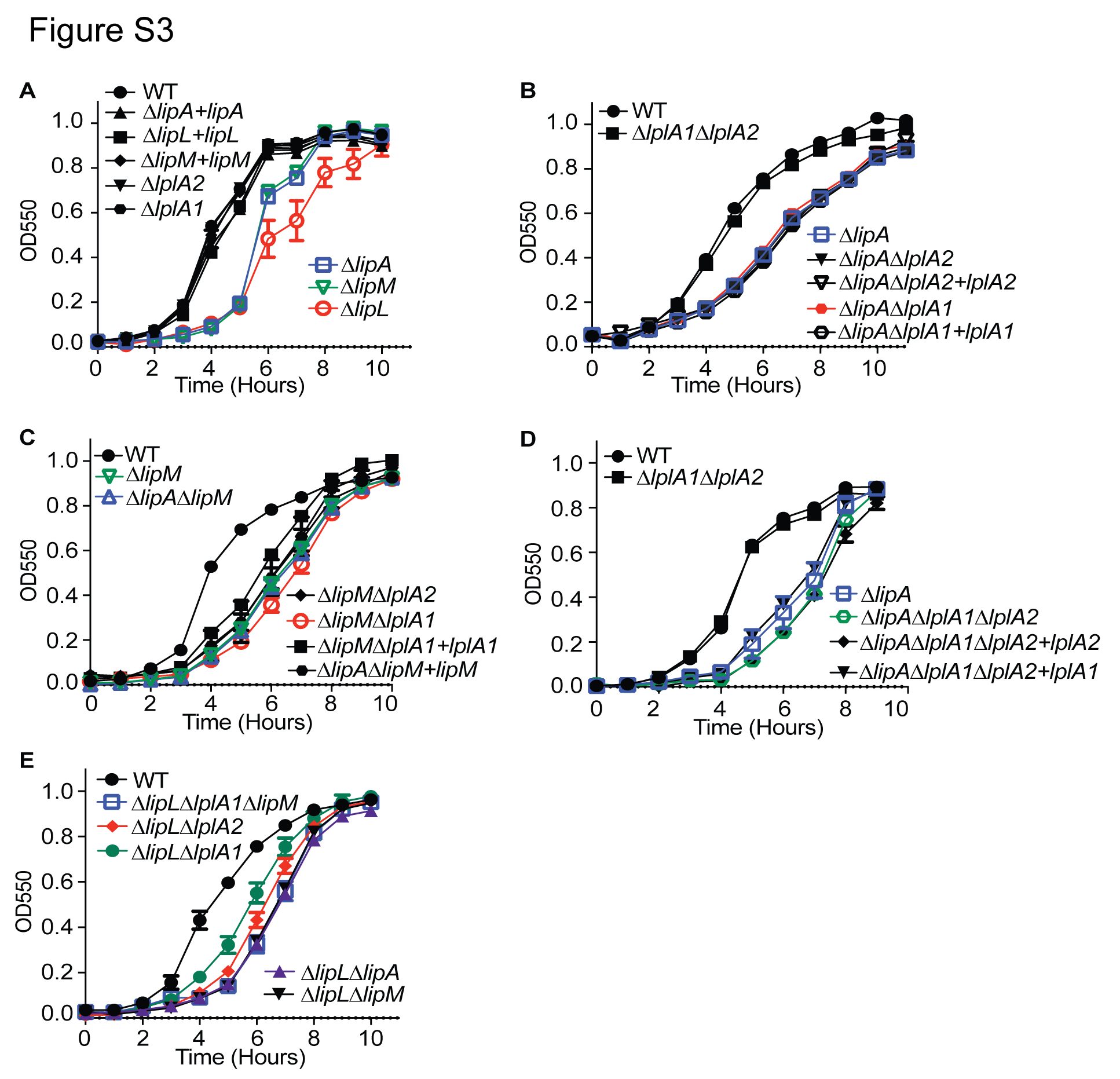

Supplement: S3 Fig — (A-E) Growth curves of the indicated strains in RPMI + BCFA (2-methyl butyric acid, isovaleric acid, isobutyric acid, and sodium acetate). In all growth curves, the mean +/- standard deviation of triplicate data points is shown. In any case where an error bar is not visible, the standard deviation was smaller than the size of the symbol used at that data point. (TIF) [file ppat.1005933.s003.tif]

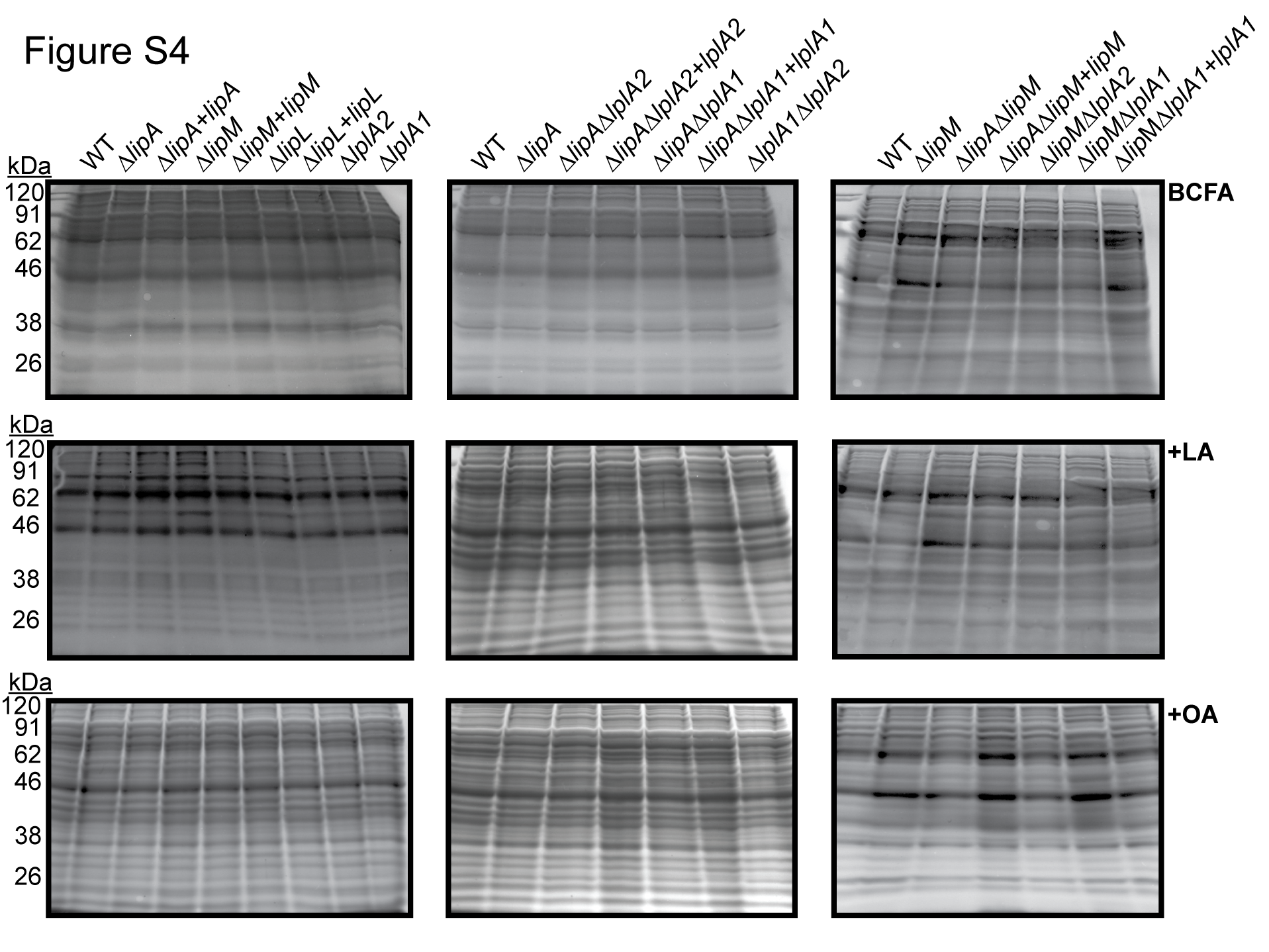

Supplement: S4 Fig — Representative coomassie-stained gels of OD normalized cell lysates of the indicated strains. Samples correspond to those used in immunoblots in Figs 2, 4 and 6. (TIF) [file ppat.1005933.s004.tif]

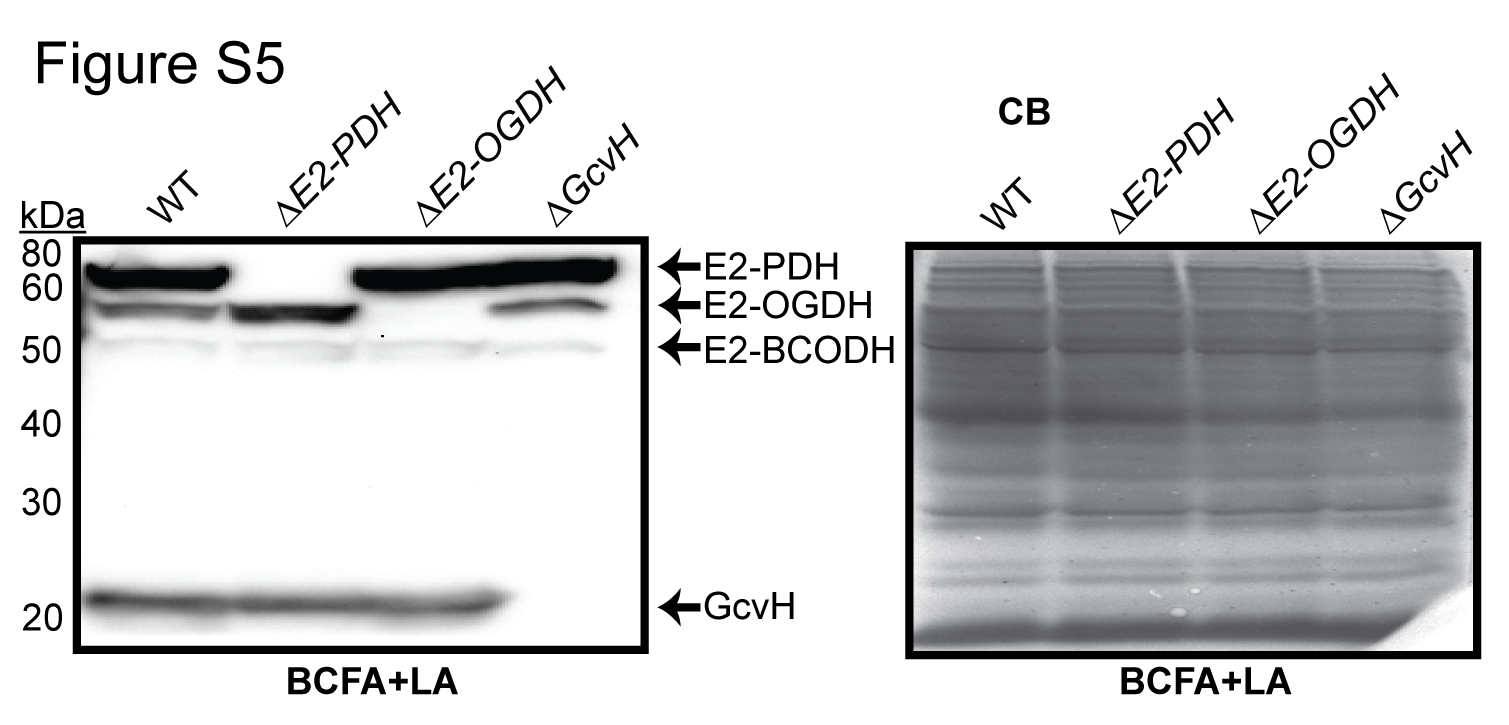

Supplement: S5 Fig — Whole cell lysates of the indicated S. aureus strains collected after 9 hours of growth in RPMI + BCFA (2-methyl butyric acid, isovaleric acid, isobutyric acid, and sodium acetate) + lipoic acid (LA), followed by immunoblotting for lipoic acid-containing proteins. (TIF) [file ppat.1005933.s005.tif]

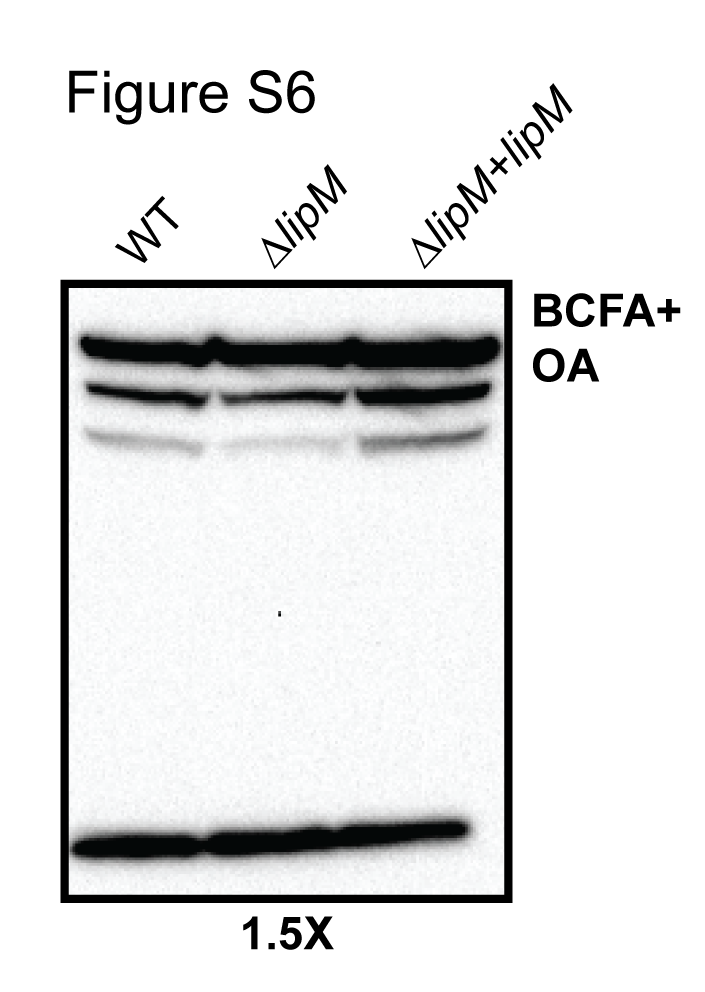

Supplement: S6 Fig — Whole cell lysates of the indicated S. aureus strains collected after 9 hours of growth in RPMI + BCFA (2-methyl butyric acid, isovaleric acid, isobutyric acid, and sodium acetate) + octanoic acid (OA), followed by loading 1.5X the amount of sample and immunoblotting for lipoic acid-containing proteins. (TIF) [file ppat.1005933.s006.tif]

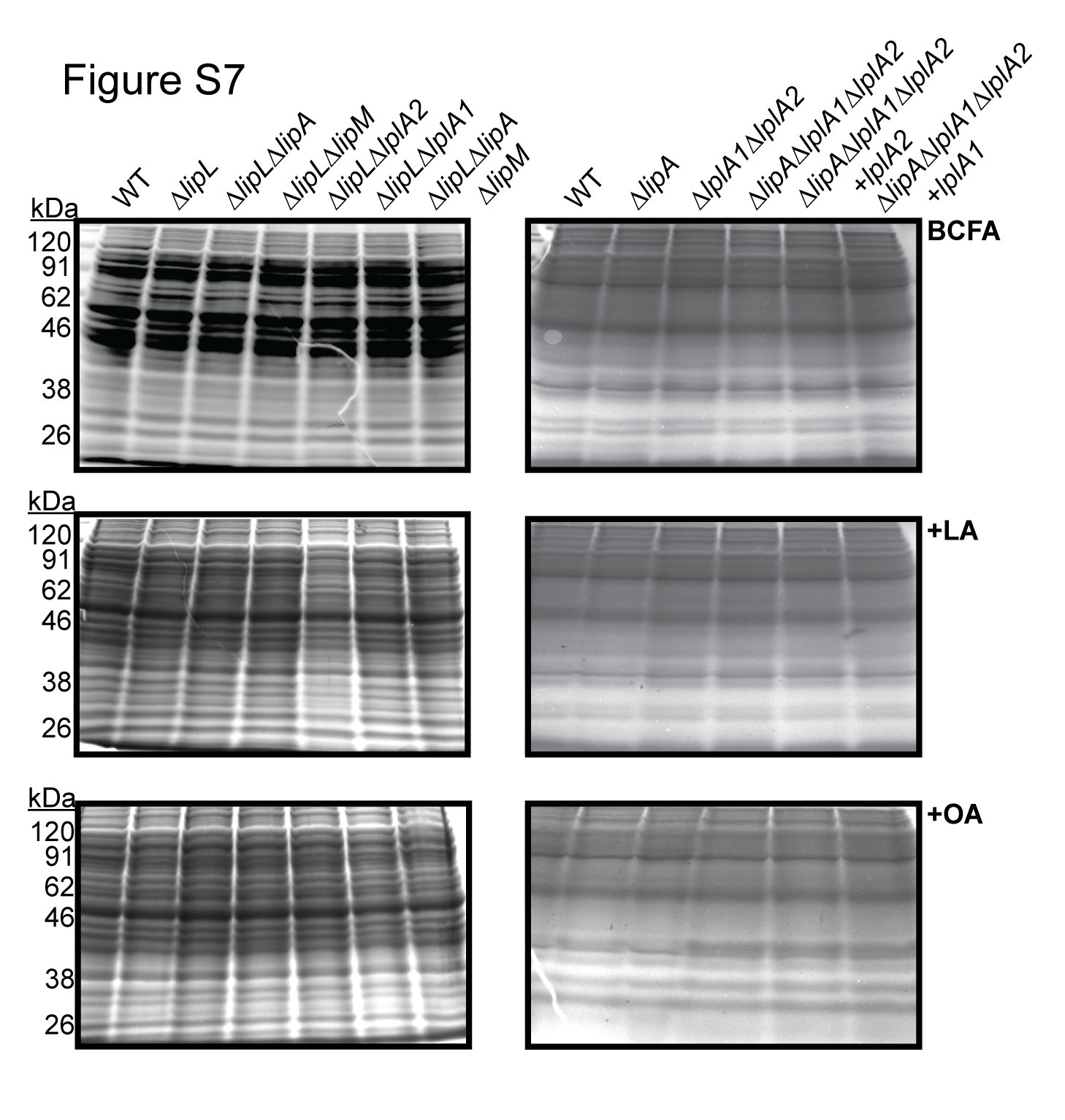

Supplement: S7 Fig — Representative coomassie-stained gel of OD normalized cell lysates of the indicated strains. Samples correspond to those used in immunoblots in Figs 7 and 8. (TIF) [file ppat.1005933.s007.tif]
